# Exosomes Derived from Human Adipose Mesenchymal Stem Cells Inhibits Fibrosis and Treats Oral Submucous Fibrosis via the miR-181a-5p/Smad2 Axis

**DOI:** 10.1007/s13770-023-00579-0

**Published:** 2023-09-27

**Authors:** Zifei Shao, Jinhao Xu, Xiaoyang Xu, Xiang Wang, Yuxi Zhou, Yiyang Li, Kun Li

**Affiliations:** 1 Department of Oral and Maxillofacial Surgery, Xiangya Stomatological Hospital and School of Stomatology, Central South University, Changsha, 410000 China; 2 Hunan Clinical Research Center of Oral Major Diseases and Oral Health, Changsha, China

**Keywords:** ADSC-Exo, OSF, Myofibroblasts, miR-181a-5p, TGF-β pathway

## Abstract

*****BACKGROUND***:**

Oral submucous fibrosis (OSF) is a chronic disease with carcinogenic tendency that poses a non-negligible threat to human health. Exosomes derived from human adipose mesenchymal stem cells (ADSC-Exo) reduces visceral and cutaneous fibroses, but their role in OSF has received little attention. The aim of this study was to investigate the effects of ADSC-Exo on OSF and elucidate the mechanism.

*****METHODS***:**

In brief, ADSCs were extracted from adipose tissues and subjected to flow cytometry and induction culture. Fibroblasts were isolated from human buccal mucosa and subjected to immunofluorescence. Myofibroblasts were obtained from fibroblasts induced by arecoline and identified. Immunofluorescence assay confirmed that myofibroblasts could take up ADSC-Exo. The effects of ADSC-Exo on the proliferative and migratory capacities of myofibroblasts were examined using the Cell Counting Kit-8 and scratch assay. Real-time quantitative polymerase chain reaction (qPCR) was performed to evaluate mothers against decapentaplegic homolog 2 (Smad2), Smad3, Smad7, collagen type 1 (Col1), Col3, alpha smooth muscle actin (α-SMA), fibronectin, and vimentin. Western blotting was performed to detect phospho (p)-Smad2, Smad2, p-Smad2/3, Smad2/3, Smad7, Col1, Col3, α-SMA, fibronectin, and vimentin. Furthermore, the dual-luciferase reporter assay was performed to prove that miR-181a-5p in ADSC-Exo directly inhibited the expression of *Smad2* mRNA to regulate the transforming growth factor beta (TGF-β) pathway. We also performed qPCR and western blotting to verify the results.

*****RESULTS***:**

ADSC-Exo could promote the proliferation and migration of myofibroblasts, reduce the expressions of p-smad2, Smad2, p-smad2/3, Smad2/3, Col1, αSMA, fibronectin, and vimentin and elevated the levels of Smad7 and Col3. In addition, miR-181a-5p was highly expressed in ADSC-Exo and bound to the 3'-untranslated region of Smad2. ADSC-Exo enriched with miR-181a-5p reduced collagen production in myofibroblasts and modulated the TGF-β pathway.

*****CONCLUSIONS***:**

ADSC-Exo promoted the proliferative and migratory capacities of myofibroblasts and inhibited collagen deposition and trans-differentiation of myofibroblasts *in vitro*. miR-181a-5p in exosomes targets Smad2 to regulate the TGF-β pathway in myofibroblasts. ADSC-Exo perform antifibrotic actions through the miR-181a-5p/Smad2 axis and may be a promising clinical treatment for OSF.

**Supplementary Information:**

The online version contains supplementary material available at 10.1007/s13770-023-00579-0.

## Introduction

Oral submucous fibrosis (OSF) is a chronic disease with malignant potential and difficult to detect early. The malignant transformation rate of OSF is approximately 4% [1]. The main clinical manifestations of OSF are restricted mouth opening, dysphagia, and even oral cancer, which seriously affect the physical and mental health of patients. Epidemiologically, the occurrence of OSF is closely related to the betel nut chewing habit. Mechanical stimulation of crude fibers and chemical stimulation, mainly through arecoline, can lead to long-term inflammation and self-repair of oral mucosal tissue, ultimately causing fibrosis [2, 3]. The betel nut chewing habit is common in densely populated parts of Asia. The number of people with the betel nut chewing habit has been growing in recent years, thereby increasing the incidence of OSF [4, 5]. The age of patients with OSF ranges widely from 8 to 80 years [6]. In the World Health Organization report, over 5 million people worldwide have OSF [7]. Therefore, the prevention and treatment of OSF are important. However, current treatments for OSF can only improve the degree of mouth opening to a certain extent and cannot reverse the pathological changes caused by OSF or yield satisfactory treatment results [8].

The development of OSF is a multistep complex process characterized by atrophy of the epidermis, vascular occlusion, and fibrous degeneration of connective tissues caused by repeated stimulations [8]. Currently, research on the development mechanism and treatment of OSF is not comprehensive. Nevertheless, regulating the transformation of fibroblasts to myofibroblasts and reducing the formation and accelerating the degradation of collagen fibers are known to be factors delaying or blocking the process of fibrosis [9, 10]. Transforming growth factor beta (TGF-β) activation in OSF stimulates the transformation of fibroblasts to myofibroblasts, accelerating collagen production and wound contraction [11, 12]. TGF-β also activates the expressions of the connective tissue growth factor and cluster of differentiation 147 to promote extracellular matrix (ECM) deposition [13, 14].

Adipose tissue-derived mesenchymal stem cells (ADSCs) can undergo multidirectional differentiation and are widely distributed in the body. They have low immunogenicity and are widely used to treat cancer [15, 16]. Exosomes are extracellular vesicles generated by the intracellular pathway. They are natural carriers containing messenger RNA (mRNA), microRNA (miRNA), proteins, and lipids that play an important role in intercellular communication [17]. TGF-β1 mRNA in exosomes released from damaged epithelial cells can activate the TGF-β pathway in other cells (e.g., fibroblasts) to promote fibrosis during epithelial–mesenchymal transition [18]. miRNAs in exosomes can regulate the translation of mRNA and inhibit the target genes widely involved in cell differentiation, proliferation, apoptosis, tissue and organ developments, and other physiological processes. miRNAs can affect the related processes of myofibroblast transformation, collagen synthesis, and ECM deposition by regulating multiple pathways in fibrotic diseases [19, 20].

ADSC exosomes (ADSC-Exo), as a carrier of miRNA, have the characteristics of easy access and low immunogenicity. They can play important roles in fat regeneration, angiogenesis, immune regulation, and wound healing through the paracrine mechanism [21–23]. Subcutaneous adipose tissue shortens the wound healing time and reduces scar formation [24]. miRNAs in exosomes participate in the pathogenesis of organ fibrosis [25–27]. Many miRNAs, such as miR-181a-5p, miR-29a-3p, miR-29b, miR-125a-5p, miR-192-5p, and miR-216a, can affect key aspects of fibrotic diseases by regulating the TGF-β pathway [27–30]. The expression of some miRNAs, such as miR-181a-5p, is high in ADSC and adipose tissue [31]. A theoretical analysis using TargetScan software revealed that miRNA-181a-5p has a binding site for Smad2, a key molecule in the TGF-β pathway. Since the TGF-β pathway promotes organ fibrosis, whether or not miR-181a-5p can treat fibrosis by regulating the Smad2/TGF-β axis should be verified.

We hypothesized that miR-181a-5p in ADSC-Exo can exert antifibrotic effects via the Smad2/TGF-β axis. Accordingly, the aim of the present study was to investigate the effect of ADSC-Exo on myofibroblasts *in vivo* and examine the possible mechanism of ADSC-Exo against fibrosis to treat OSF. We identified ADSC, ADSC-Exo, fibroblasts, and myofibroblasts. Subsequently, we investigated the effect of ADSC-Exo on myofibroblasts *in vitro* and examined the possible mechanism of ADSC-Exo against fibrosis to treat OSF. ADSC-Exo promoted the migratory and proliferative capacities of myofibroblasts and reversed the expressions of vimentin, collagen type 1 (Col1), collagen type 3 (Col3), fibronectin, and alpha smooth muscle actin (α-SMA) through arecoline. miR-181a-5p in ADSC-Exo can target and regulate Smad2 in myofibroblasts to inhibit the TGF-β signaling pathway and, thus, exert antifibrotic effects. The results of the present study enriches the application prospect of ADSC-Exo and may provide a reasonable explanation for the therapeutic strategy of ADSC-Exo for OSF.

## Materials and methods

### Patients and ethics approval

The cells (ADSC and fibroblasts) extracted from the adipose and oral buccal mucosa tissues in the experiment were obtained from the Xiangya Stomatological Hospital. All patients provided informed consent. Inclusion criteria were no systemic diseases, relevant family history, or current or recent medication. The study protocol was approved by the Ethics Committee of the Xiangya Stomatological Hospital of the Central South University. (Ref. no. 20220008).

### Isolation and culture of fibroblasts and myofibroblasts

Briefly, buccal mucosal tissues were dipped thrice with 10% penicillin–streptomycin solution (10,000 U/mL penicillin and 10 mg/mL streptomycin, BI, Kibbutz, Israel) and cut into pieces with scissors in a sterile environment. The reaction was terminated by digestion with 6 mg/mL type I collagenase (Sigma, St. Louis, MO, USA) for 20 min at 37 °C. The supernatant was centrifuged to obtain buccal mucosa-derived fibroblasts, which were cultured with 10% fetal bovine serum (FBS; BI) and 10% penicillin–streptomycin solution in a humidified incubator (5% v/v CO_2_ and 37 °C). We detected surface markers of fibroblasts, including vimentin (green, 1:200, BI) and S100A4 (red, 1:200, BI) using immunofluorescence. We expanded the extracted cells until approximately the 5^th^ sub-passages and then used them for subsequent experiments. Fibroblasts were cultured at a concentration of 2.5 × 10^5^ cells/well in six-well plates and starved in a starvation culture medium (1% FBS) for 24 h when grown to approximately 70% confluence. We stimulated it for 2 days with 20 μg/mL arecoline (Solarbio, Beijing, China). The concentration of arecoline was determined based on preliminary experimental results. Thus, we induced fibroblasts to transform to myofibroblasts. Subsequently, the experimental groups were cultured with ADSC-Exo for 2 days after 24 h of starvation culture. Finally, proteins and RNA were extracted from cells for subsequent testing.

### Isolation and identification of ADSC

Similar to the aforementioned fibroblast extraction process, after removing vascular tissues, adipose tissues were minced and digested with 4 mg/mL type I collagenase for 20 min. Subsequently, the mixture was allowed to sit for 3–5 min before removing the supernatant. The supernatant was centrifuged to obtain primary ADSC, and proliferation was continued in the culture with Medium Essential Medium alpha (MEM-α; BI) containing 10% FBS. To identify surface markers of ADSC, the fluorescence-conjugated antibodies CD105-PE, CD73-PE, CD90-PE, CD34-PE, and CD45-PE (BioLegend, San Diego, CA, USA) were incubated with three sub-passages of ADSC several times and analyzed using flow cytometer (BD Pharmingen, San Diego, CA, USA). To identify the differentiation potential, ADSCs were cultured for several days, according to the instructions of the adipogenic (Cyagen, Santa Clara, CA, USA) and osteogenic (Cyagen) induction kits. We then visualized the differentiation results after Oil Red O (Solarbio) or Alizarin Red S (Solarbio) ing according to corresponding instructions under a microscope. We then used an alkaline phosphatase (ALP) kit (BI) to detect the stem cell properties of ADSCs. Finally, a light microscopic (Ningbo ShunYu Instruments Co., Ltd., Ningbo, China) analysis was performed.

### Isolation and identification of ADSC-Exo

All steps of exosome extraction were carried out at 4 °C to ensure exosome activity. Before approximately 70% confluence, ADSCs at passages 3–6 were cultured in 100-mm cell culture dishes (ExCell Biotech Co., Ltd., Shanghai, China) with MEM-α, containing 10% FBS, 1% l-glutamine (BI), and 1% penicillin–streptomycin solution. Subsequently, the supernatant was removed before changing the extractive exosome special MEM-α, which contained 10% exosome-free serum FBS (BI), 1% l-glutamine, 100 U/mL penicillin, and 100 μg/mL streptomycin. The supernatant was then collected as an extract, and the ADSCs were subjected to a passage culture. The process was repeated until passage 6. The extract liquid was first filtered through a 0.22 µm strainer (Millipore, Billerica, MA, USA) to remove cells. Next, cell debris and apoptotic bodies were removed from the extracts (3000 relative centrifugal force [RCF], 30 min), and centrifugation was performed in the 100 KDa Ultracel-50 regenerated cellulose membrane (Millipore; 5000 RCF, 30 min), followed by adding the Total Exosome Isolation Reagent (Invitrogen, Carlsbad, CA, USA) overnight, as required. The mixture was centrifuged the next day at 5000 RCF (× g) for 30 min in the Eppendorf tubes. Finally, the supernatant in the Eppendorf tube was removed, and the resulting precipitate was exosomes. The morphology of extracted ADSC-Exo was assessed using transmission electron microscopy (TEM; FEI Tecnai G2 Spirit, Hillsboro, OS, USA), and its size and distribution were analyzed using the nanoparticle tracking analysis (NTA, Particle Metrix, Meerbusch, Germany). The expressions of CD9, CD63, TSG-101, and β-actin (Abcam, Cambridge, UK) in exosomes were detected using immunoblotting. The bicinchoninic acid protein assay kit (Thermo Fisher Scientific, Waltham, MA, USA) was used to quantify the exosome concentrations. These extracted exosomes were passed through a 0.22 μm filter again to maintain sterility before being subjected to cellular experiments. Subsequently, we labeled ADSC-Exo with the green fluorescent dye DiO (Thermo Fisher Scientific) to examine the internalization in myofibroblasts. Briefly, ADSC-Exo diluted in 200 μL phosphate-buffered saline (PBS) was incubated with DiO for 5 min, and 1 mL of PBS and 500 mL of Total Exosome Isolation Reagent were added to terminate the staining, and the mixture was centrifuged at 100,000 RCF for 90 min to obtain the successfully labeled ADSC-Exo. We stimulated myofibroblasts with ADSC-Exo (DiO-labeled) in the 1% FBS cell culture medium for 24 h. After fixing with 4% paraformaldehyde, the nucleus was counterstained with 4′,6-diamidino-2-phenylindole (DAPI; BI, Israel), and the cytoskeleton was stained with phalloidin (Abcam). Optical microscopy was performed (Leica, Germany).

### Real-time quantitative polymerase chain reaction (qPCR)

Cells were washed at least thrice with pre-chilled 4 °C PBS, and the TRIzol reagent (Sigma) was added to lyse cells for 30 min to obtain total RNA. The total RNA concentration obtained was measured (Microdrop, Norderstedt, Germany). The PrimeScript™ RT reagent kit (Enzyme, USA) was used to reverse-transcribe RNA (500 ng) into complementary DNA (cDNA). The UltraSYBR Mixture (Enzyme, USA) was used to amplify cDNA. The qPCR reaction system utilized 20 µL/well, and amplification was performed for 40 cycles as follows: pre-denaturation at 95 °C for 10 min, denaturation at 95 °C for 15 s, and annealing at 60 °C for 1 min. The relative expression was calculated using the 2^−ΔΔCT^ method, which were normalized against glyceraldehyde 3-phosphate dehydrogenase (GAPDH). We used the stem-loop method to quantify miRNAs. The reverse transcription kit supplied by EnzyArtisan (miRNA first strand cDNA synthesis kit, Shanghai, China) was used to reverse-transcribe miRNAs (800 ng) into cDNA. The miScript SYBR Green PCR kit (EnzyArtisan) and miRNA-specific primers (Sangon Biotech Shanghai Co., Ltd., Shanghai, China) were used in the amplification stage of PCR. Each reaction was conducted thrice to reduce random errors. Table 1 lists the primers obtained from Shanghai Biotechnology.

**Table 1:** The primers obtained from Shanghai Biotechnology

Name	Form	prodSize	Sequence
miR-181a-5p	FORWARD	65	AACACGCAACATTCAACGCTGT
miR-181a-5p	REVERSE	65	ATCCAGTGCAGGGTCCGAGG
miR-181a-5p	RT Primer	65	GTCGTATCCAGTGCAGGGTCCGAGGTATTCGCACTGGATACGACACTCAC
COL1A1	FORWARD	133	TGATCGTGGTGAGACTGGTCCTG
COL1A1	REVERSE	133	CTTTATGCCTCTGTCGCCCTGTTC
COL3A1	FORWARD	146	AGGGAATGCCAGGAGAAAGAGGAG
COL3A1	REVERSE	146	AGCAGGACCAGGAGGACCAATG
α-SMA/ ACTA2	FORWARD	139	CTTCGTTACTACTGCTGAGCGTGAG
α-SMA/ ACTA2	REVERSE	139	CCCATCAGGCAACTCGTAACTCTTC
smad2	FORWARD	132	CTCTTCTGGCTCAGTCTGTTAA
smad2	REVERSE	132	AAGGAGTACTTGTTACCGTCTG
smad3	FORWARD	116	AGAGAGTAGAGACACCAGTTCT
smad3	REVERSE	116	GAAGTTAGTGTTTTCGGGGATG
smad7	FORWARD	224	ATGTTCAGGACCAAACGATCT
smad7	REVERSE	224	GGATGGTGGTGACCTTTGG
Fibronectin 1	FORWARD	82	AATAGATGCAACGATCAGGACA
Fibronectin 1	REVERSE	82	GCAGGTTTCCTCGATTATCCTT
Vimentin	FORWARD	97	CCTTCGTGAATACCAAGACCTGCTC
Vimentin	REVERSE	97	AATCCTGCTCTCCTCGCCTTCC

### Western blotting

To extract cellular proteins, fibroblasts and myofibroblasts were collected, washed at least thrice with PBS at 4 °C, and solubilized in the cell lysis radioimmunoprecipitation assay buffer (Beyotime Biotechnology, Shanghai, China) containing the proteinase inhibitor phenylmethylsulfonyl fluoride (Boster Bio, Wuhan, China) and phosphatase inhibitors (BI). After the cells were lysed on ice for 30 min, the mixture was centrifuged to remove cell debris (4 °C, 12,000 rpm, 20 min). The bicinchoninic acid protein assay was used to measure the protein concentration. Depending on the target protein, 6–11% sodium dodecyl-sulfate polyacrylamide gel electrophoresis gels (Beyotime Biotechnology) and polyvinylidene difluoride transfer membranes (Millipore) were selected for electrophoresis, and the total protein (20 μg) was transferred after the sodium dodecyl-sulfate loading buffer (BI) treatment. After the membranes were blocked for 2 h in 5% bovine serum albumin (BI, Israel) diluted by tris-buffered saline with 0.1% Tween 20 (BI) at room temperature (about 25 °C), we incubated them with primary antibodies specific to Col1 (1:1000, Abcam), Col3 (1:1000, Abcam), α-SMA (1: 1000, Abcam), CD9, CD63 (1:1000, BI), Smad2 (1:800, BI), p-Smad2 (1:800, BI), Smad2/3 (1:1000, BI), p-Smad2/3 (1:1000, BI), Smad7 (1:1000, BI), fibronectin (1:1000, BI), GAPDH (1:2000, BI), and β-actin (1:2000, BI) at 4 °C overnight. The next day, horseradish peroxidase-conjugated anti-rabbit IgG secondary antibodies (1:10,000, BI) and an enhanced chemiluminescence kit (BI) were to visualize the immunoreactive traces at room temperature using the Fluorochem system (Tanon, Shanghai, China). Finally, the expression intensity of the proteins and normalized against GAPDH were analyzed using ImageJ software.

### Wound scratch and cell counting kit-8 (CCK-8) assays

Fibroblasts were inoculated into 6-well plates and induced to become myofibroblasts at 70% confluence with 20 µg/mL arecoline. When fibroblasts and myofibroblasts grew to 100% confluence, the single cell layer was scraped with a 200 μL sterile pipette tip to create a wound gap, and the suspended cells were washed away with PBS before adding the serum-free medium for culture. Subsequently, ADSC-Exo at 50 and 100 μg/mL were added to the specific myofibroblasts for induction. The migration areas of cells in scratch experiments at 12, 24, 36, 48, and 60 h were statistically analyzed using ImageJ software. Similarly, to detect the cell proliferation capacity, we examined the optical density (BioTek, Winooski, VT, USA) of fibroblasts and myofibroblasts with ADSC-Exo added at 1, 2, and 3 days using the CCK-8 kit (BI). Subsequently, we calculated the cell activity and performed statistical analyses.

### Validation of the regulatory relationship between miR-181a-5p and SMAD2 mRNA

We used the HEK293 cell line to verify that miR-181a-5p targeted SMAD2 mRNA. We first examined the expression of miR-181a-5p and SMAD2 mRNA in miR-181a-5p and control mimics using qPCR. We further examined the expression of the SMAD2 protein in cells after promoting or inhibiting miR-181a-5p using western blotting. Finally, the dual-luciferase reporter (DLR) assay was used to demonstrate that Smad2 is a direct target of miR-181a-5p. Briefly, wild-type or mutant Smad2 cells were co-transfected with miR-181a-5p or control mimics using the Lipofectamine™ 3000 transfection reagent (L3000015, Invitrogen). After 24 h, we harvested samples for luciferase assays (CKX41, Olympus, Tokyo, Japan).

### Statistical analysis

Statistical analyses were performed using SPSS 17.0, and data were plotted using GraphPad Prism 8. Student’s *t* test and analysis of variance (one-way ANOVA) were used to process the corresponding data. Every experiment was performed at least thrice. Data are expressed as mean ± standard error. A *p* value < 0.05 was considered to indicate statistical significance.

## Results

### Identification of ADSCs and ADSC-Exo

**Fig. 1:**
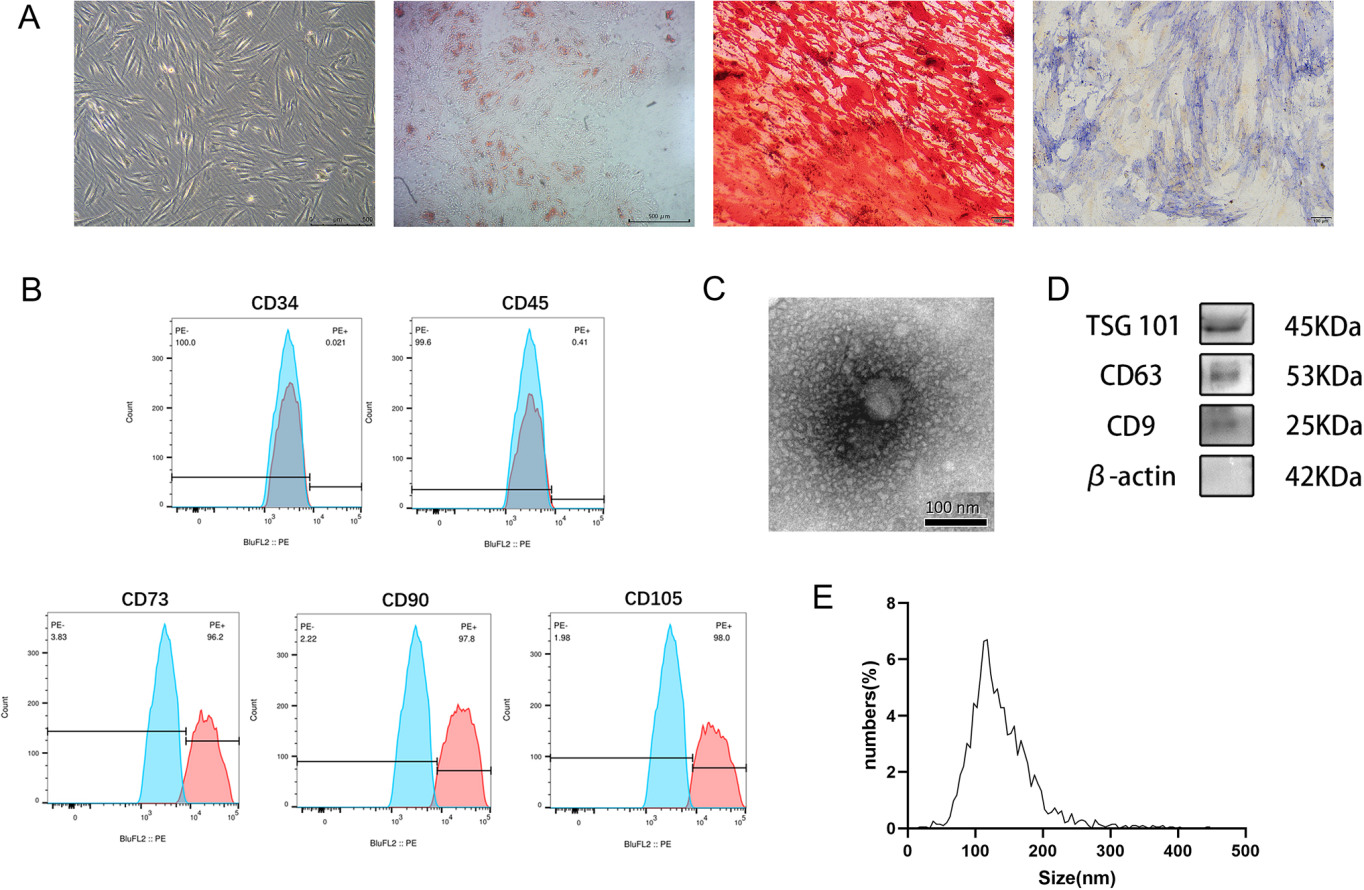
Identification of ADSCs and ADSC-Exo. **A** ADSC (scale bars = 500 μm), Oil Red O staining (scale bars = 500 μm), Alizarin Red S staining (scale bars = 100 μm), and ALP staining (scale bars = 100 μm) were shown separately using optical morphology. **B** In the flow cytometric assay of ADSCs, the extracted ADSC surface did not express CD34 or CD45 and significantly overexpressed the mesenchymal stem cell surface marker proteins CD73, CD90, and CD105. **C** Transmission electron microscopy of ADSC-Exo reveals the bowl-like structure of the clear film, scale bar = 100 nm. **D** Western blotting results of ADSC-Exo surface (CD9, CD63, and TSG 101) and negative (β-actin) markers. **E** NTA results of the particle diameter and distribution of the extracted ADSC-Exo

The extracted ADSCs showed a typical fibroblast-like spindle morphology in culture, and a swirling pattern of cell arrangement was observed in the culture dish. After stimulating ADSCs with the corresponding induction medium, we saw distinct red lipid droplets within cells and red osteogenic tissues stained with Oil Red O and Alizarin Red S, indicating that the ADSCs we obtained had multiple differentiation potential. The Alkaline Phosphatase (ALP) assay of ADSCs also indicated that the extracted ADSCs had good stemness (Fig. 1A). Subsequently, we examined the surface markers of these cells using flow cytometry, which showed high expressions of CD73 (PE), CD90 (PE), and CD105 (PE) and negative results for CD73 (PE) and CD90 (PE) (Fig. 1B). The multidirectional differentiation potential of cells and correct surface markers demonstrated our success in obtaining ADSCs. Furthermore, the morphology of ADSC-Exo presented a bowl-like structure of the clear film on TEM (Fig. 1C). NTA showed that the average particle diameter of the purified ADSC-Exo was within 100–150 nm (138.7 nm; Fig. 1E), and western blotting for exosome surface (CD9, CD63, and TSG101) and negative (β-actin) markers were as expected (Fig. 1D). These data suggest that our purified nanoparticles are consistent with the definition of exosomes.

### Identification of myofibroblasts and myofibroblast uptake in ADSC-Exo experiments

**Fig. 2:**
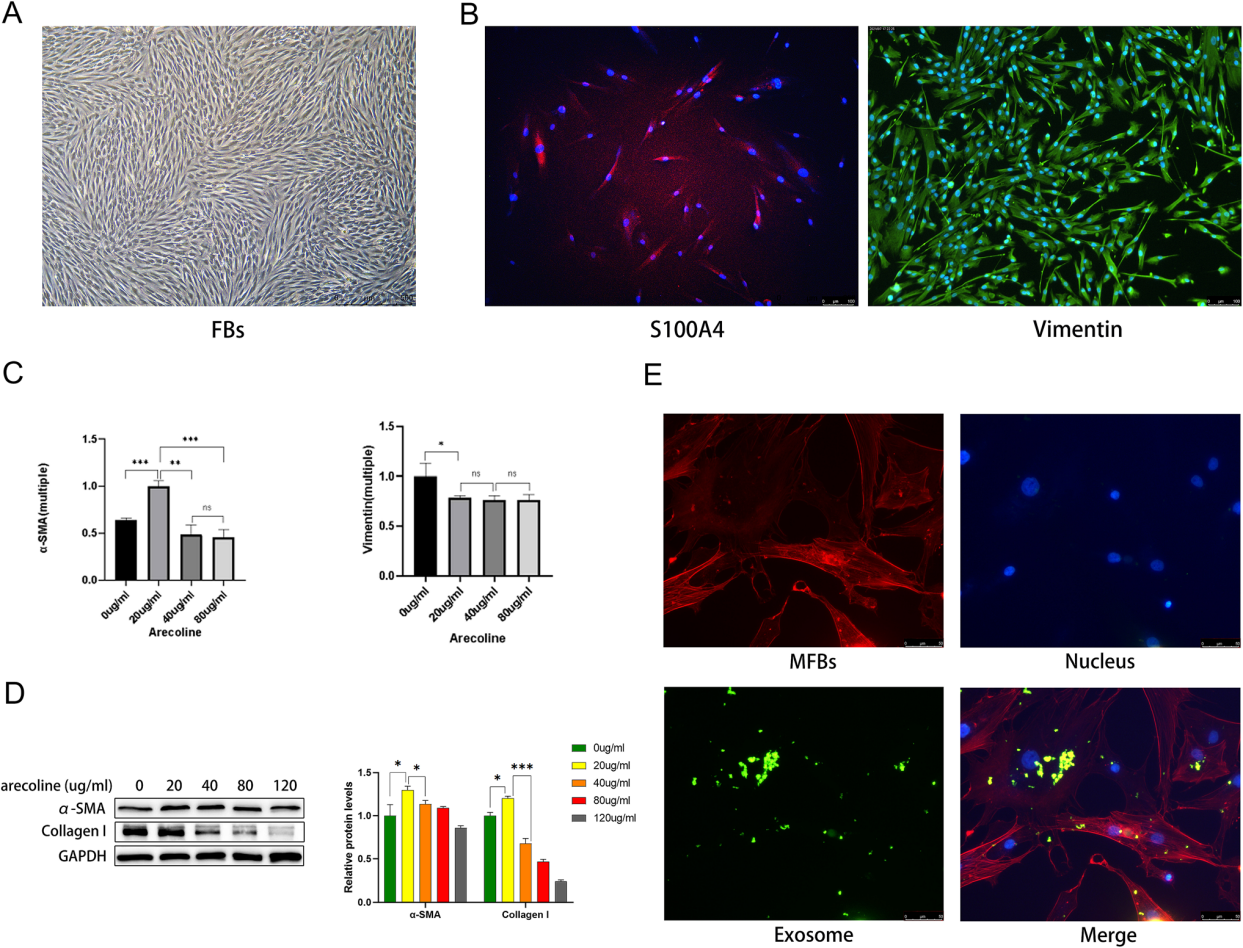
Identifying myofibroblasts and myofibroblast uptake ADSC-Exo experiments. **A** Optical morphology of fibroblasts under a microscope, scale bars = 500 μm. **B** Fibroblasts (blue nucleus, DAPI) expressing S100A4 (red) and vimentin (green) under an immunofluorescence microscope, scale bars = 100 μm. **C** Changes in vimentin and α-SMA-related mRNA after treatment of fibroblasts with different concentrations of arecoline. **D** Changes in intracellular α-SMA and Col1 protein expression after stimulation of fibroblasts with different concentrations of arecoline in western blotting. **E** After uptake by myofibroblasts (red cytoskeleton, Phalloidine), exosomes (green, DiO) are distributed around the nucleus (blue, DAPI). (*p < 0.05; **p < 0.01, ***p < 0.001)

Under the light microscope, fibroblasts showed a typical spindle shape, and densely growing fibroblasts showed a vortex distribution in the cell culture dish (Fig. 2A). Immunofluorescence microscopy also showed that fibroblasts expressed S100A4 (red) and vimentin (green), which are surface marker proteins of fibroblasts (Fig. 2B). These features prove that we extracted the typical fibroblasts. Real-time PCR showed that the expression level of α-SMA, a key protein of myofibroblasts, was the highest at 20 µg/mL of arecoline, while the expression of vimentin, a marker protein of fibroblasts, decreased after adding at least 20 µg/mL of arecoline (Fig. 2C). High arecoline concentrations lead to reduction in Col1, which may be related to arecoline dose-related cytotoxicity. Western blotting also showed that the highest protein expressions of α-SMA and Col1 were induced by arecoline at a concentration of 20 µg/mL, indicating that the appropriate concentration of arecoline can induce the conversion of fibroblasts to myofibroblasts (Fig. 2D). In DiO-labeled ADSC-Exo co-cultured with myofibroblasts, ADSC-Exo were taken up by myofibroblasts, and ADSC-Exo was mainly distributed near the nucleus after entering the cell (Fig. 2E). This proved that ADSD-Exo could enter and function in myofibroblasts.

### ADSC-Exo can promote the cell viability and migration of myofibroblasts and reduce the fibrosis of OSF

**Fig. 3:**
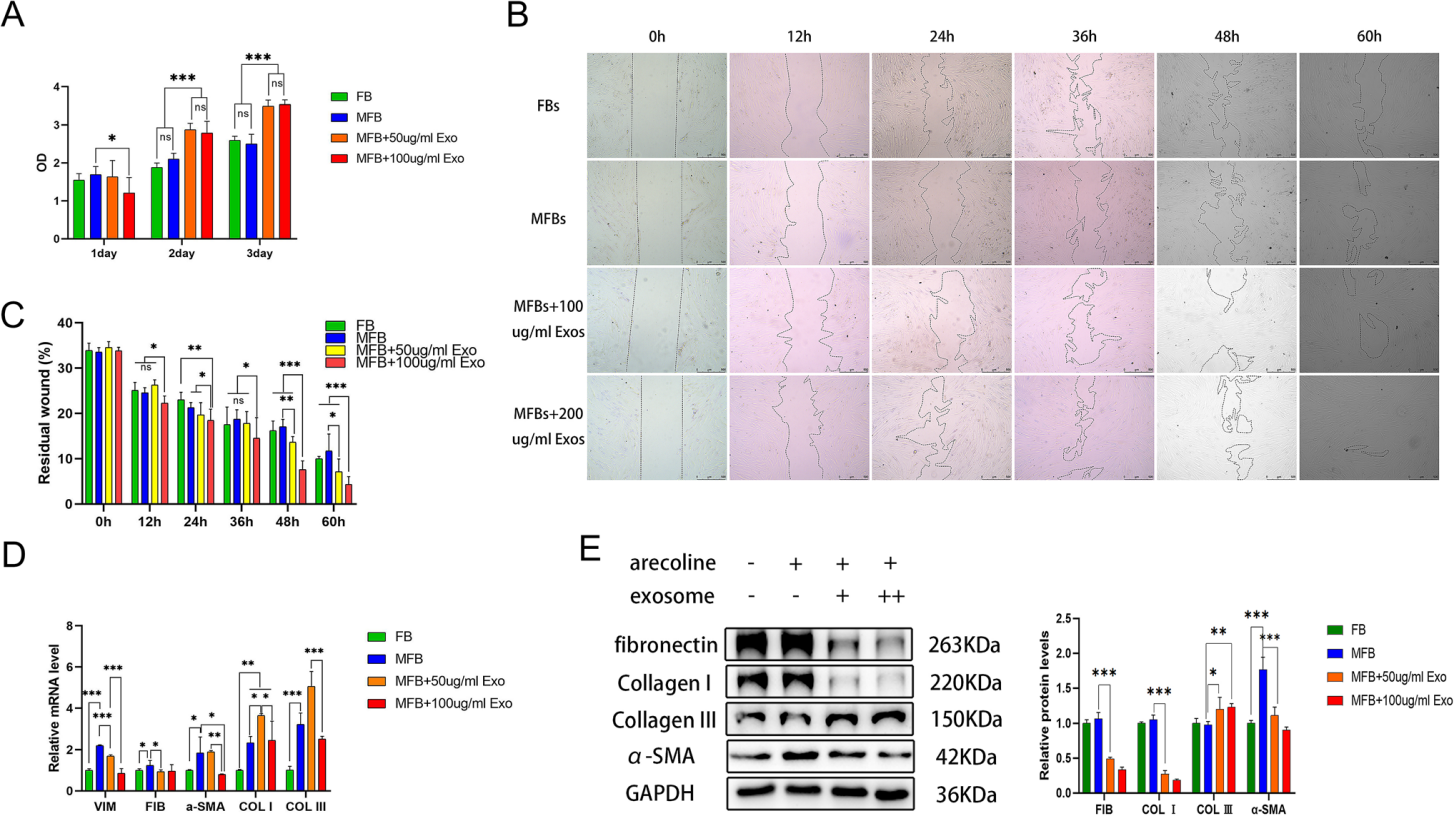
ADSC-Exo can promote the cell viability and migration of myofibroblasts and reduce the fibrosis of OSF. **A** In the CCK-8 assay, optical density represents the cell proliferation capacity. **B** In the scratch assay, the area of the remaining scratch is marked with a dashed line, which is inversely proportional to the cell migration capacity, scale bars = 500 μm. **C** After 48 h, ADSC-Exo could significantly promote the cell migration ability. **D** Detection of fibrosis-related mRNA expressions of vimentin, fibronectin, Col1, Col3, and α-SMA using qPCR. **E** Western blotting results of the expression of fibrosis-related proteins fibronectin, Col1, Col3, and α-SMA. (*p < 0.05; **p < 0.01, ***p < 0.001)

To explore the effect of ADSC-Exo on myofibroblasts, we stimulated fibroblasts to become myofibroblasts with 10 µg/mL arecoline and then added ADSC-Exo. The cell viability was detected using CCK-8 at 1, 2, and 3 days. ADSC-Exo showed slight inhibition on day 1 but significantly promoted cell proliferations on days 2 and 3. Since the proliferation capacities of myofibroblasts with ADSC-Exo added at 2 and 3 days were significantly stronger than those of fibroblasts and myofibroblasts, we collectively concluded that ADSC-Exo could promote cell proliferation. Low concentrations of arecoline (10 µg/mL) did not affect the proliferative capacity of cells (Fig. 3A). The scratch test showed that ADSC-Exo significantly promoted the migration ability of myofibroblasts after 48 h. High concentrations of ADSC-Exo (100 µg/mL) exerted the migration promoting effect at 12 h (Fig. 3B, C). In addition, real-time PCR showed that ADSC-Exo reversed the high expressions of vimentin, fibronectin, Col1, Col3, and α-SMA mRNA in myofibroblasts. However, in myofibroblasts spiked with low concentrations of ADSC-Exo (50 µg/mL), Col1 and Col3 showed a transient increase, possibly suggesting that their changes are not monotonically decreasing during regulation (Fig. 3D). The corresponding western blotting showed that ADSC-Exo significantly reduced the protein expressions of fibronectin, Col1, and α-SMA and reversed the transformation of fibroblasts into myofibroblasts induced by arecoline (Fig. 3E). Altogether, these findings suggest that ADSC-Exo promotes the proliferation and migration abilities and alleviates the transformation of myofibroblasts.

### miR-181a-5p in ADSC-Exo exerts an anti-fibrotic effect on myofibroblasts by directly inhibiting Smad2 mRNA

**Fig. 4:**
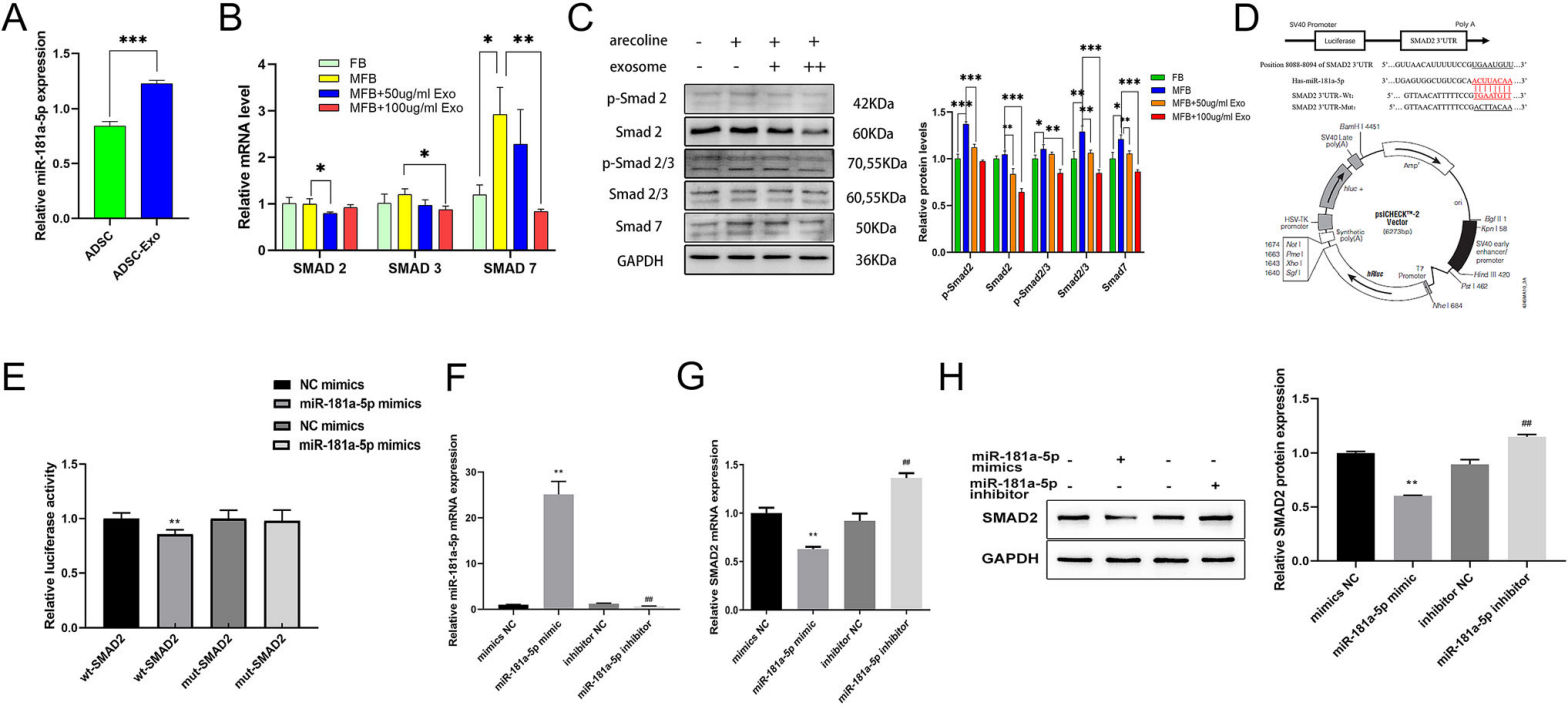
miR-181a-5p inhibits the TGF-β pathway by directly suppressing the Smad2 expression. **A** Detection of the miR-181a-5p expression in ADSC and ADSC-Exo by qPCR. **B** qPCR results of the effects of ADSC-Exo on Smad2, Smad3, and Smad7 on myofibroblasts **c** In western blotting, ADSC-Exo reversed the expression of key proteins of the TGF-β signaling pathway, including Smad2, Smad2/3, and Smad7 in myofibroblasts. **D** Theoretical binding sites predicted by TargetScan 7.0 and schematic diagram of the DLR plasmid construction. **E** Fluorescence changes in DLR assays. **F, G** Expressions of miR-181a-5p and Smad2 was detected using qPCR in the experimental groups of miR-181a-5p mimic and miR-181a-5p inhibitor as well as in their control groups *in vitro*. **H** Expression of the Smad2 protein after miR-181a-5p was overexpressed *in vitro* (*p < 0.05, **p < 0.01, ***p < 0.001)

**Fig. 5:**
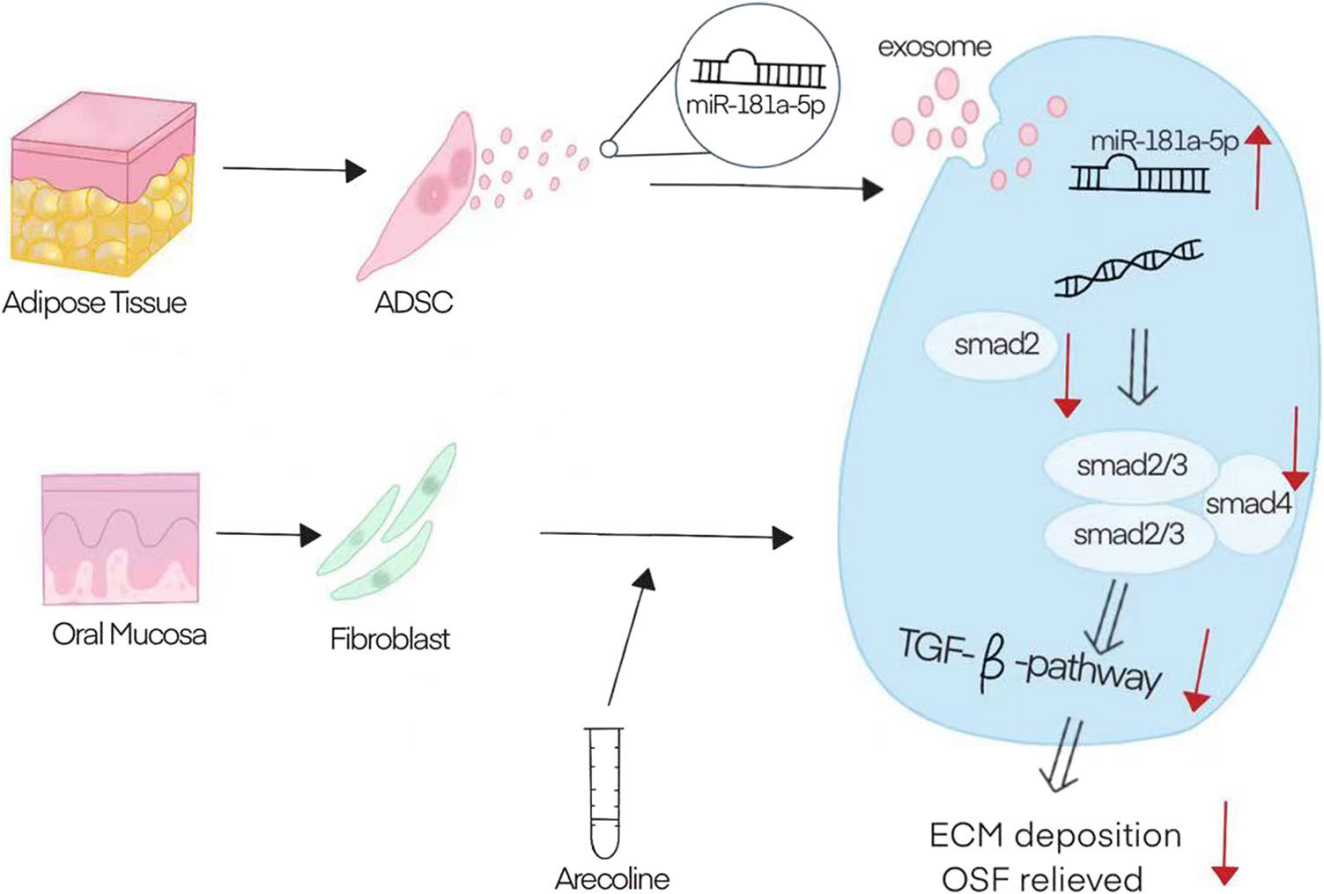
Exosomes derived from human adipose mesenchymal stem cells inhibits fibrosis and treats oral submucous fibrosis via the miR-181a-5p/Smad2 axis

qPCR showed that the miR-181a-5p concentration was higher in ADSC-Exo than in ADSC (*p* < 0.001; Fig. 4A). Smad2 and Smad3 expressions in myofibroblasts were reduced after adding human ADSC-Exo (*p* < 0.05). The Smad7 expression also decreased (*p* < 0.01; Fig. 4B). Western blotting showed that miR-181a-5p-enriched ADSC-Exo reduced the expressions of Smad2 and Smad2/3, the key proteins of the TGF-β pathway in myofibroblasts and then decreased their phosphorylations, ultimately inhibiting the TGF-β pathway (Fig. 4C). MiRNAs can bind to the 3′-untranslated region (UTR) of some mRNAs to inhibit their translation and, thus, regulate the gene expression. Using this property of miRNAs, we used TargetScan 7.0 to show that miR-181a-5p contains a theoretical binding site for the Smad2 3′-UTR. We constructed the DLR plasmid to validate this theoretical binding site (Fig. 4D). Compared to the negative control group, the miR-181a-5p mimic group significantly reduced the luciferase activity in HEK293 cells, which was transfected with a reporter plasmid containing the Smad2 wild-type 3′-UTR sequence (*p* < 0.01), while this inhibition was eliminated in the mutant Smad2 (Fig. 4E). qPCR also demonstrated a significant decrease in the Smad2 mRNA expression in cells that successfully overexpressed miR-181a-5p. Conversely, the Smad2 mRNA expression was elevated in cells that inhibited miR-181a-5p (*p* < 0.01; Fig. 4F, G). The expression results of the Smad2 protein in WB were the same as those in qPCR (*p* < 0.01; Fig. 4H). As shown in this diagram, these data indicated that miR-181a-5p in ADSC-Exo could downregulate the TGF-β activity via the miR-181a-5p/Smad2 axis (Fig. 5).

## Discussion

In this study, ADSC-Exo promoted the proliferative and migratory abilities of myofibroblasts, inhibited collagen deposition in myofibroblasts, thereby exerting an anti-fibrotic effect on the development of OSF. Further, ADSC-Exo treated the fibrosis of OSF by directly inhibiting Smad2 via miR-181a-5p. The results suggest that the anti-fibrosis molecular mechanism of ADSC-Exo is achieved via the miR-181a-5p/Smad2 axis. This study provides sufficient evidence for the possible therapeutic effects of ADSC-Exo.

Exosomes are biogenic vesicles of approximately 100 nm. Endosomes interact with other intracellular vesicles and organelles to produce the final contents of exosomes [32]. Exosomes act as a paracrine signaling mediator to influence fibrotic disease progression by transferring fibrosis-associated miRNAs to target cells [33]. They are a good miRNA carrier that can protect miRNAs from degradation by ribonuclease [34]. MiRNas are a small class of endogenous non-coding RNAs, whose main function is to downregulate the expression of target genes by binding to their 3′-UTRs [35].

ADSC-Exo, with its easy access and weak immunogenicity, can play important roles in adipose regeneration, angiogenesis, immunomodulation, and wound healing [21, 22]. During skin wound healing, ADSC-Exo can promote fibroblast proliferation and migration by acting on the PI3K/Akt signaling pathway, effectively improving collagen deposition, promoting angiogenesis, and reducing skin scar production [36]. This also explains the exosomes promoting the proliferative and migratory abilities of myofibroblasts in our experimental results. Some previous studies and our preliminary high-throughput sequencing technology showed that 45 miRNAs (including miR-181a-5p) were highly expressed in ADSCs-Exo. The Kyoto Encyclopedia of Genes and Genomes analysis revealed that the highly expressed miRNAs were related to the insulin, mitogen-activated protein kinase (MAPK), and TGF-β pathways, which are mainly involved in regulating lipogenic differentiation, vascular regeneration, and other processes [22]. Therefore, intervening and treating the development of OSF with ADSC-Exo utilizing the miR-181a-5p-rich property of ADSC-Exo are reasonable and feasible. Taking advantage of the weak immunogenicity and low ethical risk of ADSC, the modified ADSC and ADSC-Exo have a wide application prospect in treating OSF.

Fibrosis is accompanied by histopathological changes in tissues and organs, and this pathological fibrosis can occur in many organs, such as the kidneys, liver, lungs, cardiovascular system, skin, etc. By affecting the function of different organs, fibrosis often leads to serious complications and even death [37]. Approximately 600 million people worldwide chew betel nut, and OSF caused by betel nut is a chronic insidious disease with cancerous tendency, which seriously affects the physical and mental health of patients. The development of OSF is closely related to mechanical and chemical (mainly arecoline) stimulations of the coarse fibers of betel nut [2, 3]. The basic feature of fibrosis is the excessive aggregation of extracellular interstitial components, such as collagen and fibronectin, around damaged or inflamed tissue [38]. TGF-β is a key molecule of fibrosis in various organs and tissues. Biological processes, such as the cell growth cycle, cell differentiation, immune microenvironment, and ECM deposition are regulated by the TGF-β pathway [39, 40]. In the classical TGF-β signaling pathway, the cell-secreted TGF-β complex is converted to an inactive state. The inactive molecule binds to TGF-βRII and activates and phosphorylates TGF-βRI (ALK5). Subsequently, phosphorylation-activated ALK5 induces phosphorylations of the Smad2 and Smad3 proteins to p-Samd2/3 in the cytoplasm. Finally, p-Samd2/3 forms a complex with Smad4 and accumulates in the nucleus to regulate the transcription of fibrosis-related genes [41–43].

The present results showed that ADSC-Exo can reduce the expressions of Smad2 and Smad3 proteins by significantly downregulating Smad2 and Smad3 mRNAs, which decrease the amounts of phosphorylation activations of Smad2 and Smad3 and finally reduce the activity of the TGF-β pathway. Furthermore, the myofibroblast uptakes of the ADSC-Exo and DLR assays showed that ADSC-Exo inhibit the activity of the TGF-β signaling pathway by targeting Smad2 via miR-181a-5p. However, in addition to the decreased expression of Samd2 at the mRNA and protein levels, the Smad3 expression decreased, suggesting that our theory may not be comprehensive. In addition, Smad7 can play a negative regulatory role by competitively binding phosphorylated ALK5 with SMAD2/3 [44, 45]. In myofibroblast, Smad7 can inhibit Smad-dependent and Smad-independent TGF-β responses and fibrosis through an endogenous TGF-β-induced negative feedback mechanism [46]. Our qPCR results were consistent with the western blotting results in that fibroblasts were activated by TGF-β after arecoline stimulation, and activating the negative cellular feedback mechanism increased the Smad7 expression. In contrast, ADSC-Exo prevented the over-activation of the TGF-β pathway, thereby reversing the elevation in the Smad7 expression. This result also demonstrated that ADSC-Exo are effective in regulating the TGF-β pathway to treat fibrosis.

The diverse composition of exosome contents includes nucleic acids, proteins, lipids, amino acids, and metabolites [32]. Although we demonstrated that the miR-181a-5p concentration was higher in ADSC-Exo than in ADSC, other substances in ADSC-Exo may also play a regulatory role in alleviating OSF. For example, miR-192-5p in ADSC-Exo alleviates hypertrophic scar fibrosis [30]. ADSC-Exo can treat liver fibrosis and alleviate non-alcoholic fatty liver disease by delivering miR-223-3p [47]. miR-181a-5p prevents liver fibrosis by activating autophagy in hepatic stellate cells [27]. In contrast, ADSC-Exo inhibited ultraviolet-mediated photoaging of skin fibroblasts by upregulating the p-Smad2/3 expression [48]. This difference is related to the resting and activated states of fibroblasts.

A complex regulatory network of the TGF-β-related signaling pathways is jointly involved in the development of fibrosis. In addition to the classical TGF-β pathway, many proteins are involved, such as ALK1/Smad1/5, JAK, STAT3, and PI3K [49]. The PI3K/Akt/mTOR signaling pathway controls cell differentiation and is closely related to autophagy [50, 51]. The link between fibrotic disease and autophagy is also increasingly studied. Autophagy is inhibited in fibrotic lung tissue, but miR-449a can activate autophagy via the TGF-β1/ERK/MAPK/Bcl2 axis, thereby attenuating the development of pulmonary fibrosis [52]. Curcumin activates peroxisome proliferator-activated receptor alpha to reduce reactive oxygen species levels and oxidative stress and increases glutathione levels in hepatocytes, affecting the downstream AMPK/PI3K/AKT/mTOR axis, ultimately promoting autophagy activation and inhibiting liver fibrosis [53]. Activating autophagy in tubular epithelial cells promotes TGF-β degradation, reduces mature TGF-β secretion, and ultimately reduces collagen production in interstitial cells and attenuates renal fibrosis [54]. The relationship between autophagy and OSF has also been explored, with early OSF showing activation of autophagy, while intermediate and late OSF showing reduced autophagy [55]. However, a more in-depth study on the detailed relationship between autophagy and TGF-β in OSF has not been performed. Autophagy plays an important role in regulating intracellular energy and material homeostasis. In fibrotic tissues, because Col1 is resistant to all mammalian collagenases, it may play a key role in fibrotic disease by interfering with ECM remodeling [56]. In our experimental results, ADSC-Exo successfully reversed the high expression of Col1 in myofibroblasts and inhibited fibrosis. However, the reduction in Col3 was not significant, and the qPCR results showed that low concentrations (50 µg/mL) of ADSC-Exo could not reduce the expression of collagen-related mRNA. These results suggest that the process of ADSC-Exo in regulating collagen production is complex and variable. Autophagy is an important way for cells to regulate protein expression, and autophagy interacts with the TGF-β signaling pathway to jointly regulate the development of OSF. Exploring the specific mechanisms of the TGF-β signaling pathway and autophagy in OSF explains the development of OSF and can be a therapeutic target for OSF. Our study demonstrated that ADSCs-Exo can alleviate OSF via the miR-181a-5p/Smad2 axis, but the mechanism of ADSCs-Exo for OSF treatment remains to be explored more comprehensively in depth. Our protocol using ADSC-Exo for the OSF treatment would be a promising research direction.

In conclusion, in the study, ADSC-Exo was effective in promoting the proliferation and migratory capacities of myofibroblasts and reversing the collagen deposition and myofibroblast trans-differentiation caused by betaine on fibroblasts. Furthermore, we verified that miR-181a-5p in ADSC-Exo could directly target Smad2 to regulate the TGF-β pathway to alleviate arecoline-induced OSF. Our study provides a new therapeutic strategy and elucidates the specific mechanisms underlying the clinical treatment of OSF with ADSC-Exo.

### Supplementary Information

Below is the link to the electronic supplementary material.

Supplementary file1 (PDF 1513 kb)

## Data Availability

All data generated or analysed during this study are included in this published article [and its supplementary information files].
