# Supplementary material for: Exosomes Derived from Human Adipose Mesenchymal Stem Cells Inhibits Fibrosis and Treats Oral Submucous Fibrosis via the miR-181a-5p/Smad2 Axis
Source: Tissue Eng Regen Med. 2023 Sep 27;21(1):123–35. doi: 10.1007/s13770-023-00579-0 (PMC10764695; doi:10.1007/s13770-023-00579-0)

Supplementary Material

TITLE

Exosomes derived from human adipose mesenchymal stem cells inhibits fibrosis and treats oral submucous fibrosis via the miR-181a-5p/Smad2 axis.

Author

Zifei Shao[1], Kun Li*

* Corresponding author, Email: 406889138@qq.com

Western blot

Identification of ADSC-Exo

ACTIN

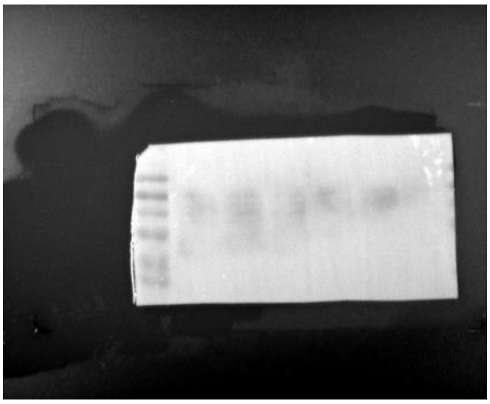

CD9

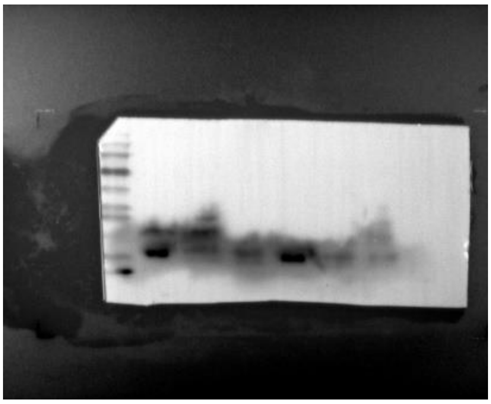

CD63

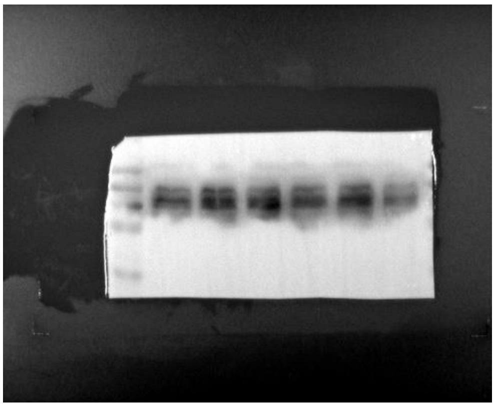

TSG101

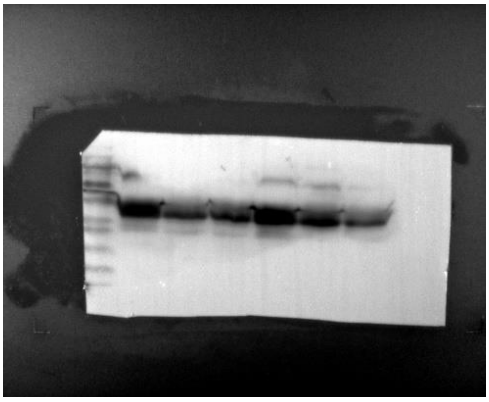

1,2,3: Exosomes derived from adipose tissue (AT-Exo)

4,5,6: Exosomes derived from adipose-derived stem cells (ADSC-Exo)

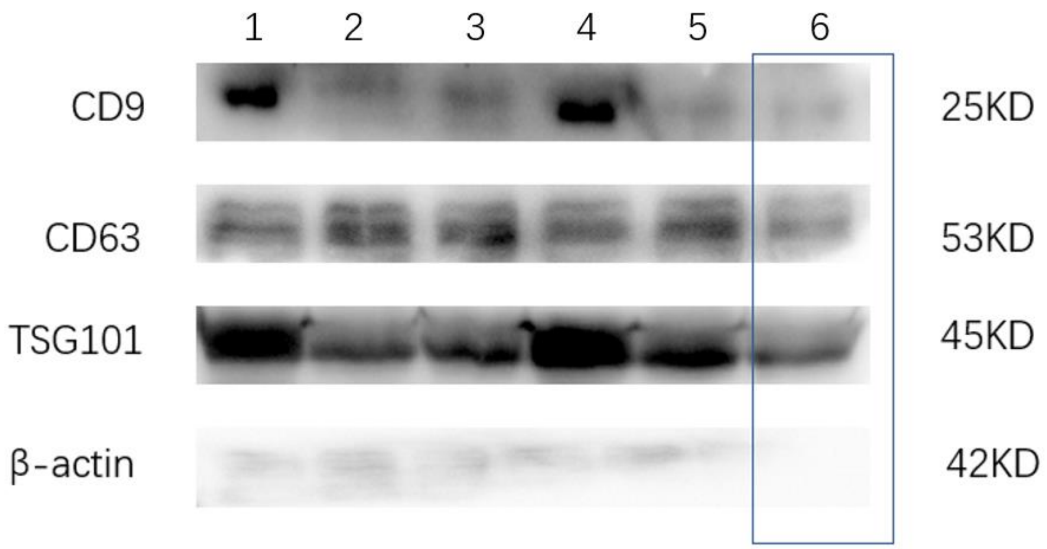

Identification of MFB

Protein： GAPDH

Samples of each column from left to right：

1. Control

2. Arecoline

3. Arecoline + 50ug/ml ADSC-Exo

4. Arecoline + 100ug/ml ADSC-Exo

5. Arecoline + 200ug/ml ADSC-Exo

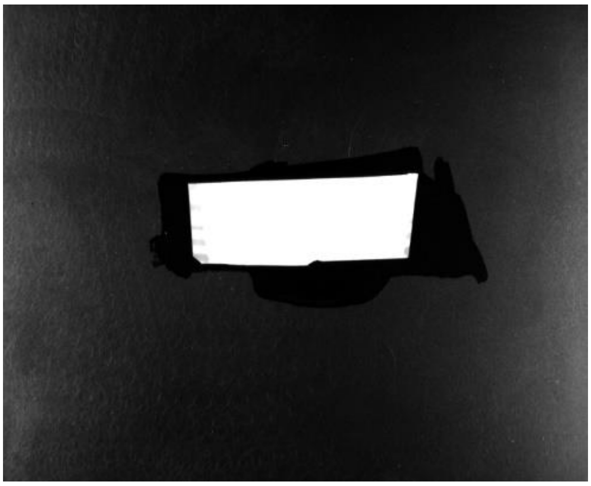

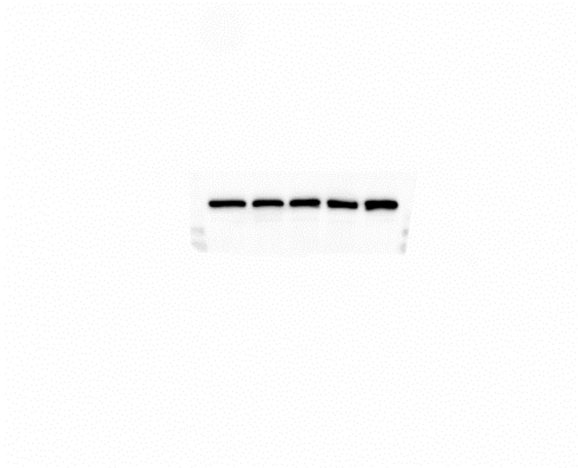

Protein: α-SMA

Samples of each column from left to right:

1. Control

2. Arecoline

3. Arecoline + 50ug/ml ADSC-Exo

4. Arecoline + 100ug/ml ADSC-Exo

5. Arecoline + 200ug/ml ADSC-Exo

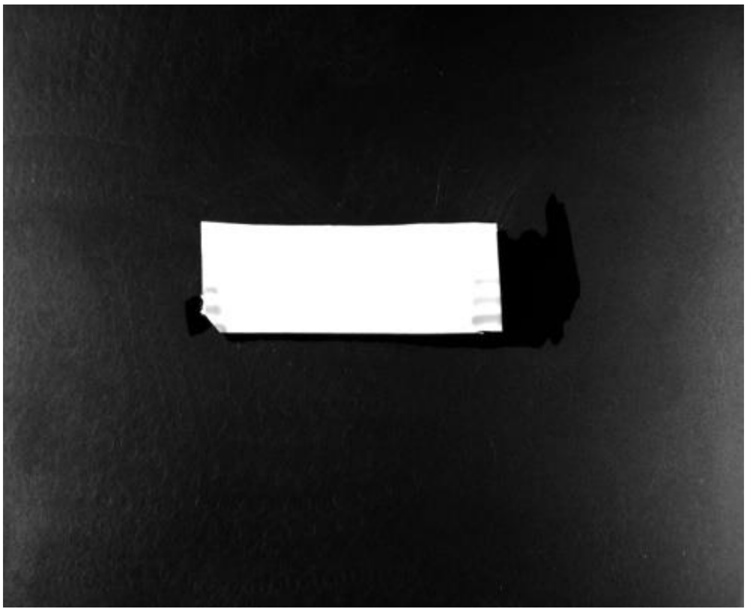

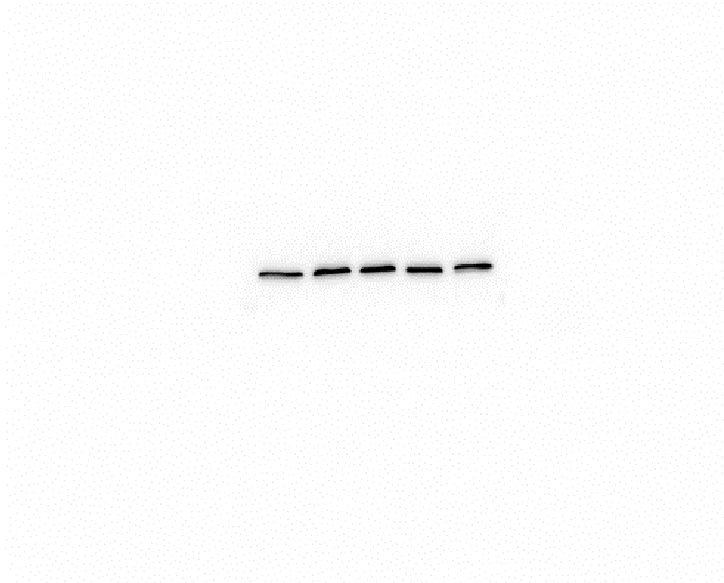

Protein：　Collagen I

Samples of each column from left to right：

1. Control

2. Arecoline

3. Arecoline + 50ug/ml ADSC-Exo

4. Arecoline + 100ug/ml ADSC-Exo

5. Arecoline + 200ug/ml ADSC-Exo

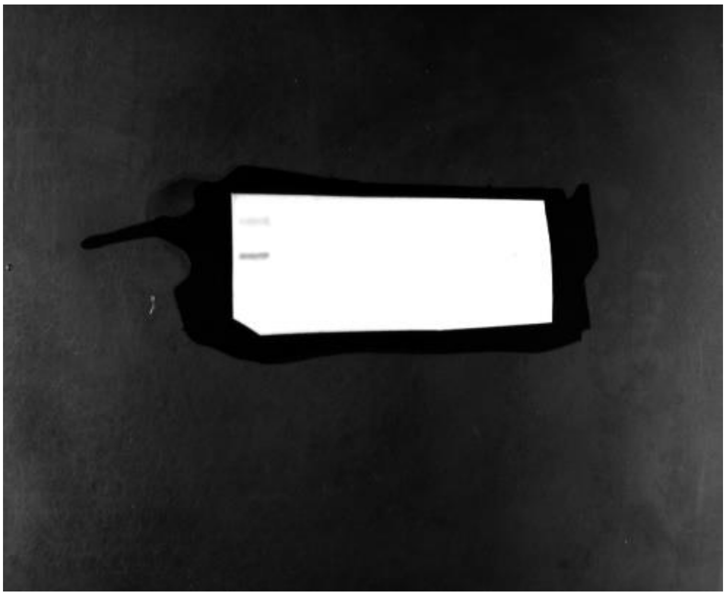

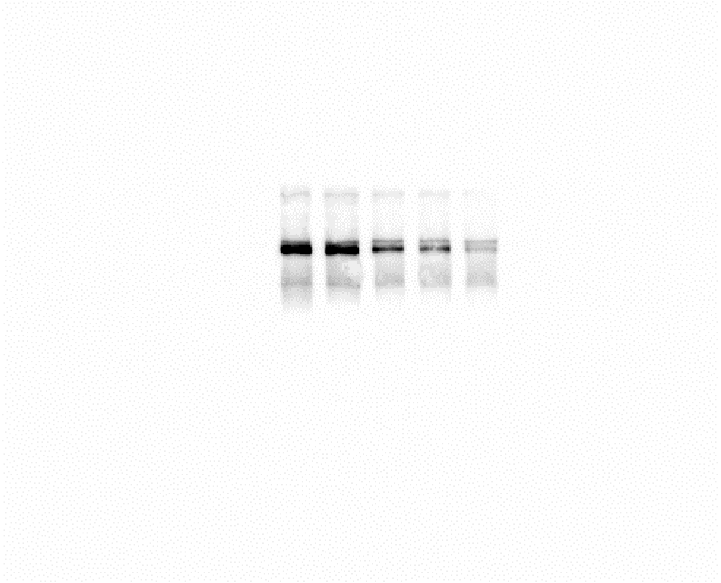

ADSC-Exo inhibits MFB via the miR-181a-5p/Smad2 axis

Protein： Collagen I

Samples of each column from left to right：

1. Arecoline + 200ug/ml ADSC-Exo

2. Arecoline + 100ug/ml ADSC-Exo

3. Arecoline + 50ug/ml ADSC-Exo

4. Arecoline

5. Control

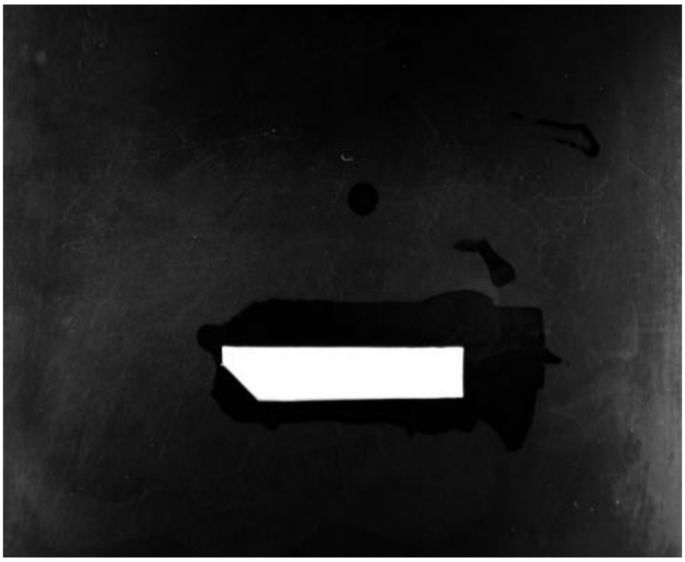

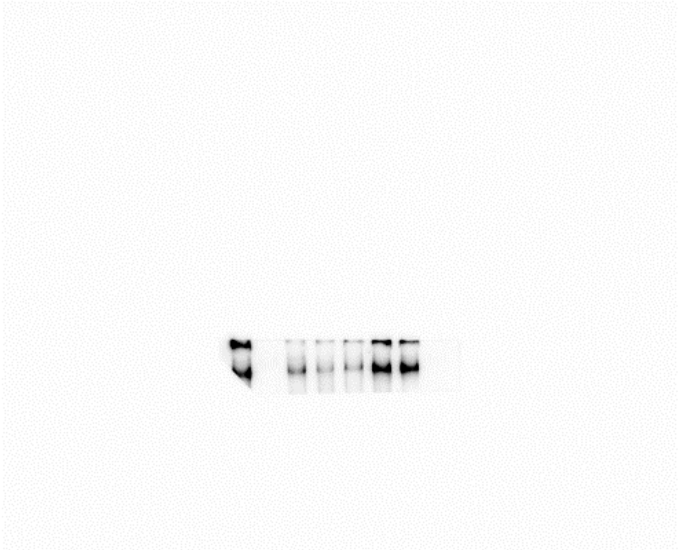

Protein： Collagen III

Samples of each column from left to right：

1. Arecoline + 200ug/ml ADSC-Exo

2. Arecoline + 100ug/ml ADSC-Exo

3. Arecoline + 50ug/ml ADSC-Exo

4. Arecoline

5. Control

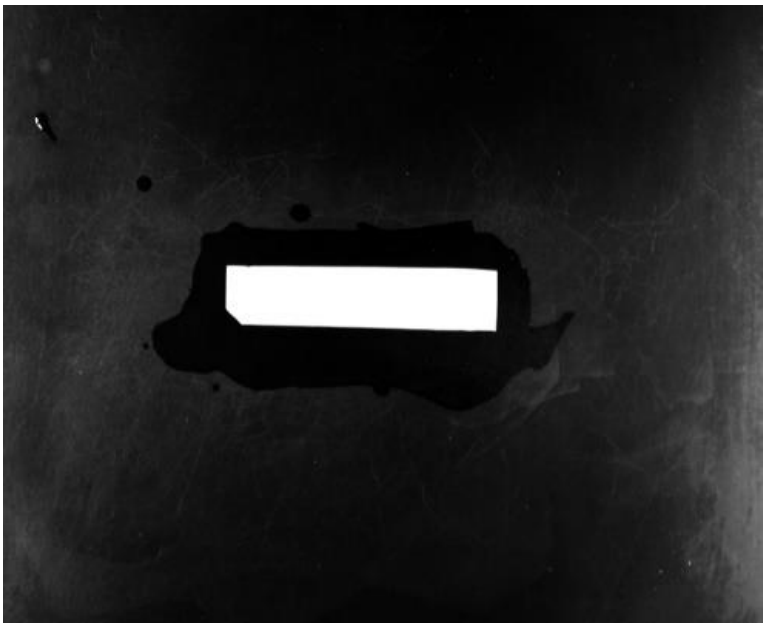

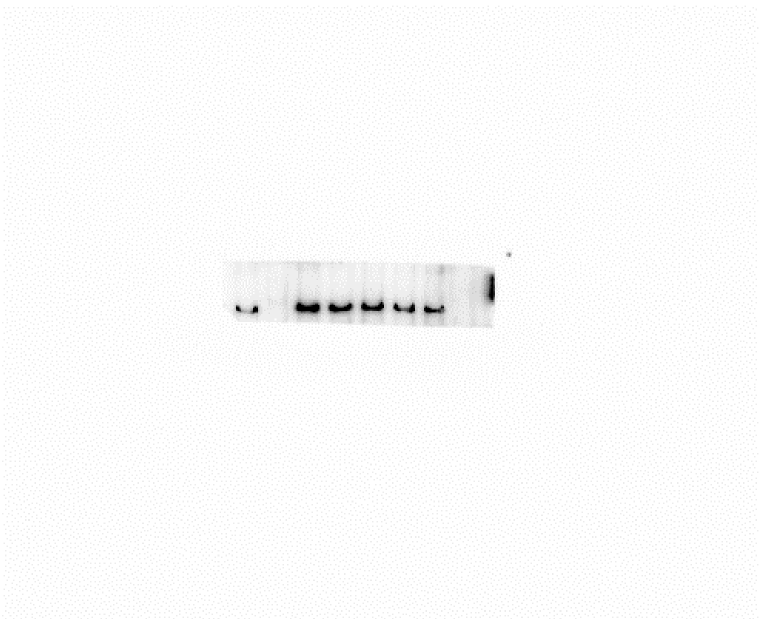

Protein： Fibronectin

Samples of each column from left to right：

1. Arecoline + 200ug/ml ADSC-Exo

2. Arecoline + 100ug/ml ADSC-Exo

3. Arecoline + 50ug/ml ADSC-Exo

4. Arecoline

5. Control

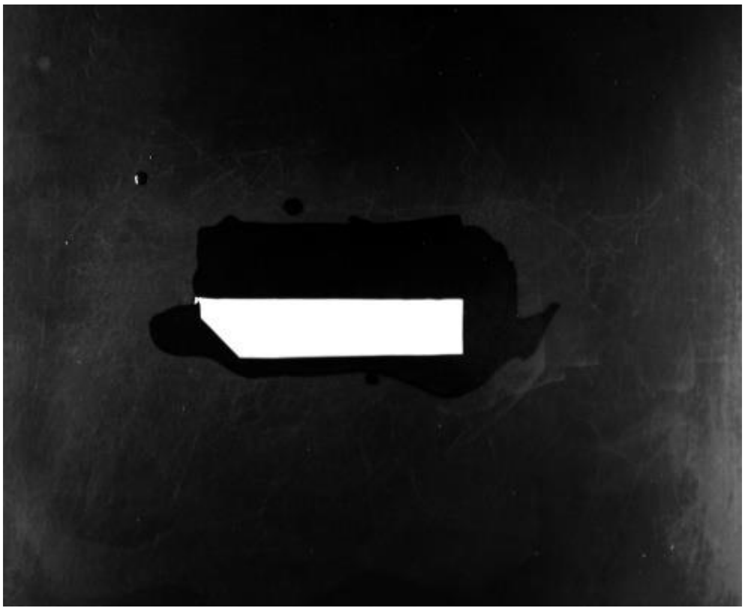

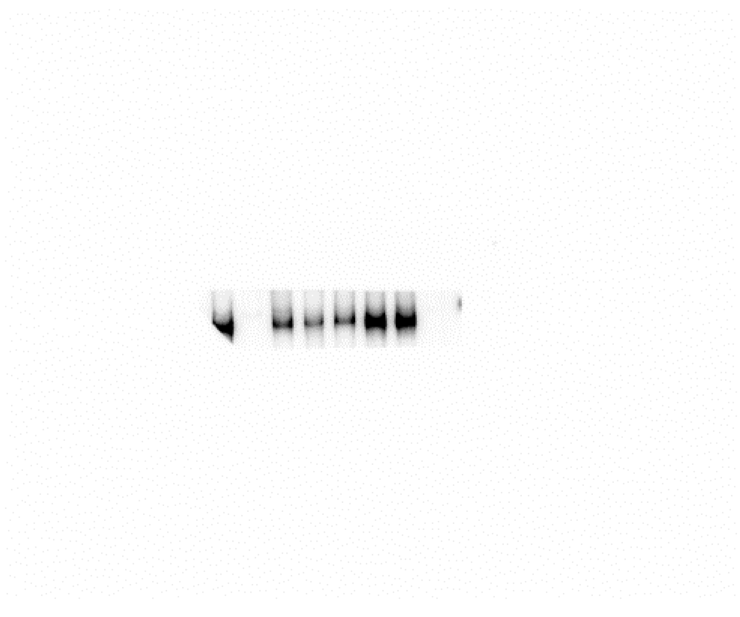

Protein： GAPDH

Samples of each column from left to right：

1. Arecoline + 200ug/ml ADSC-Exo

2. Arecoline + 100ug/ml ADSC-Exo

3. Arecoline + 50ug/ml ADSC-Exo

4. Arecoline

5. Control

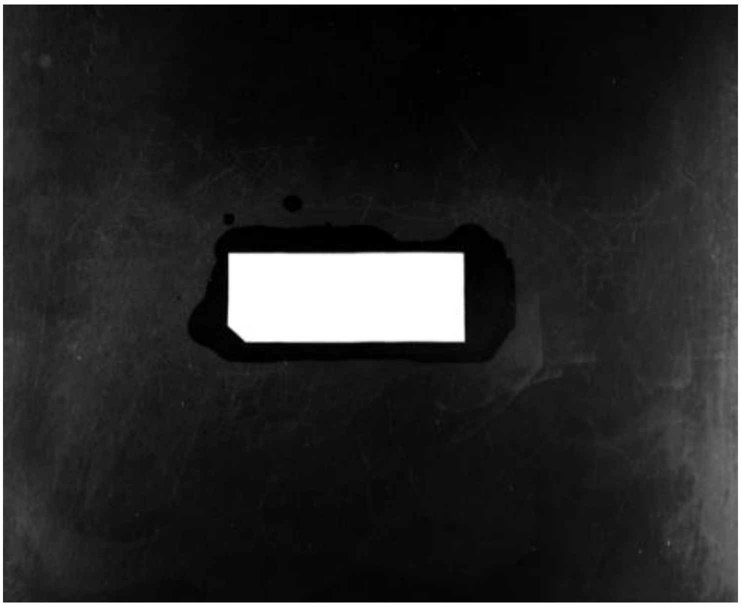

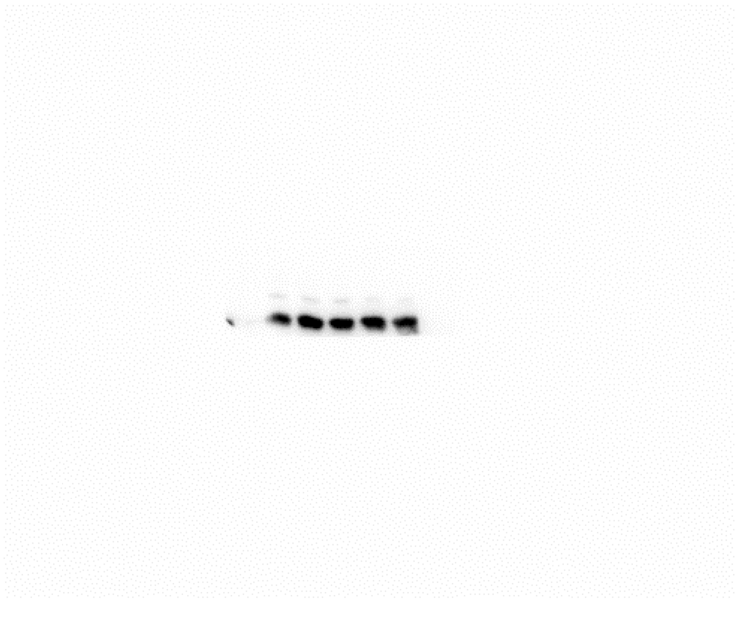

Protein： α-SMA

Samples of each column from left to right：

1. Arecoline + 200ug/ml ADSC-Exo

2. Arecoline + 100ug/ml ADSC-Exo

3. Arecoline + 50ug/ml ADSC-Exo

4. Arecoline

5. Control

6. Arecoline + 200ug/ml ADSC-Exo

7. Arecoline + 100ug/ml ADSC-Exo

8. Arecoline + 50ug/ml ADSC-Exo

9. Arecoline

10. Control

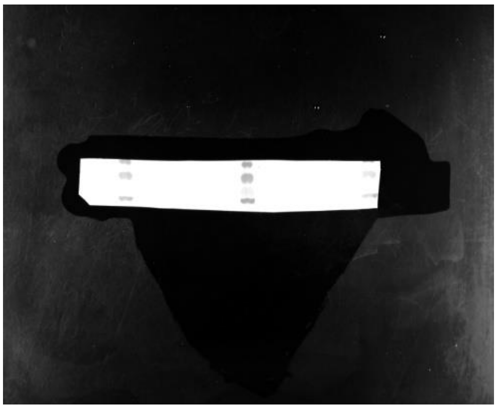

Protein：GAPDH

Samples of each column from left to right：

1. Arecoline + 200ug/ml ADSC-Exo

2. Arecoline + 100ug/ml ADSC-Exo

3. Arecoline + 50ug/ml ADSC-Exo

4. Arecoline

5. Control

6. Arecoline + 200ug/ml ADSC-Exo

7. Arecoline + 100ug/ml ADSC-Exo

8. Arecoline + 50ug/ml ADSC-Exo

9. Arecoline

10. Control

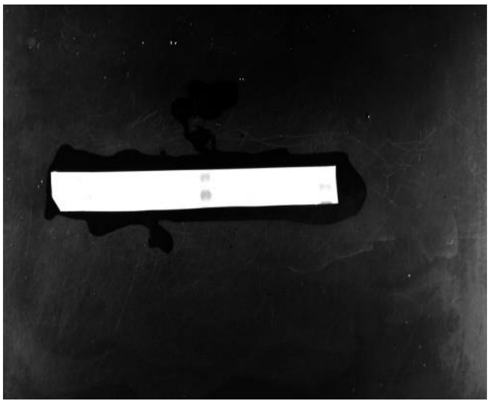

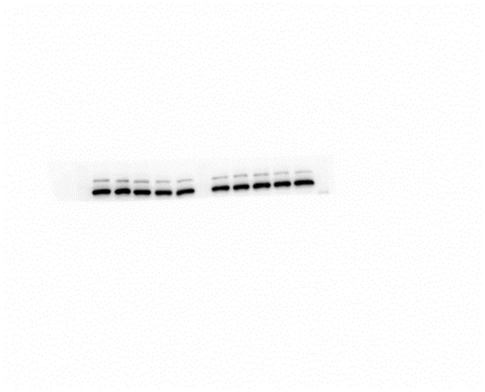

Protein： p-Smad2/3

Samples of each column from left to right：

1. Arecoline + 100ug/ml ADSC-Exo

2. Arecoline + 50ug/ml ADSC-Exo

3. Arecoline

4. Control

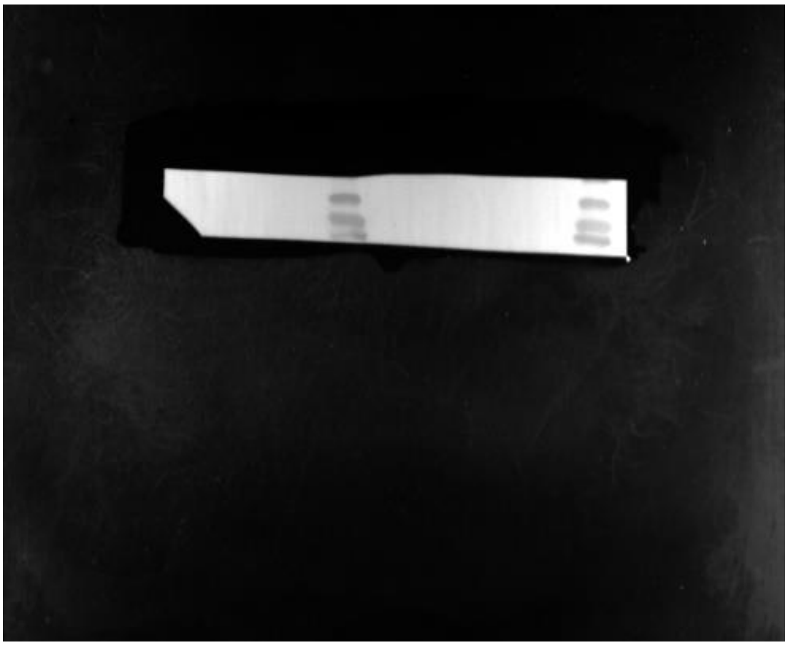

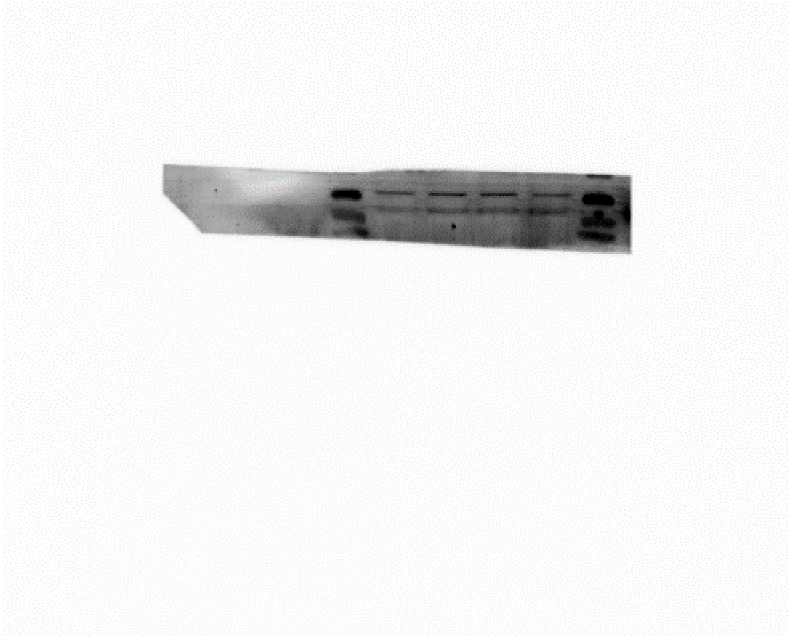

Protein： GAPDH

Samples of each column from left to right：

1. Arecoline + 100ug/ml ADSC-Exo

2. Arecoline + 50ug/ml ADSC-Exo

3. Arecoline

4. Control

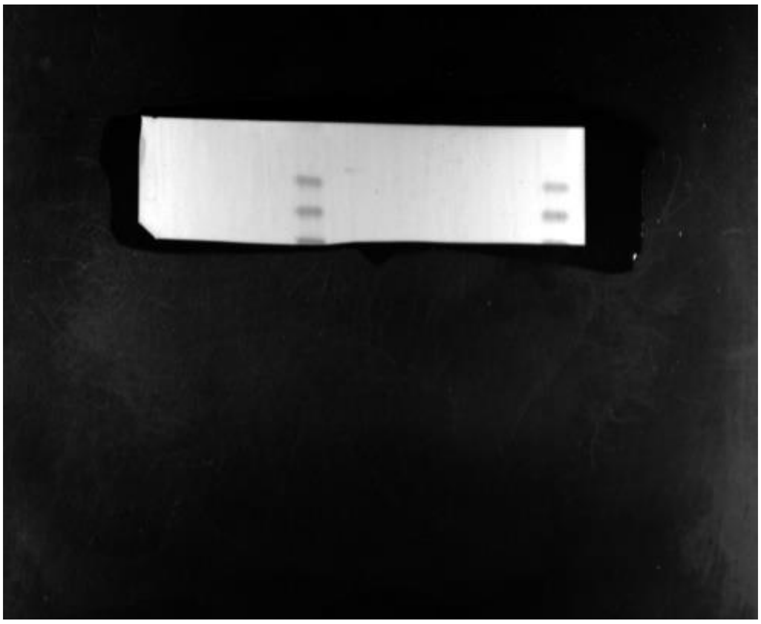

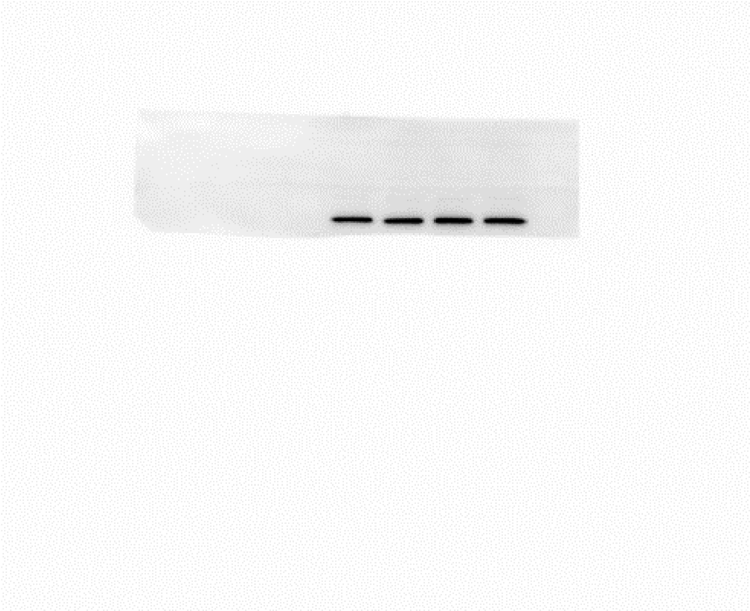

Protein： p-Smad2

Samples of each column from left to right：

1. Arecoline + 100ug/ml ADSC-Exo

2. Arecoline + 50ug/ml ADSC-Exo

3. Arecoline

4. Control

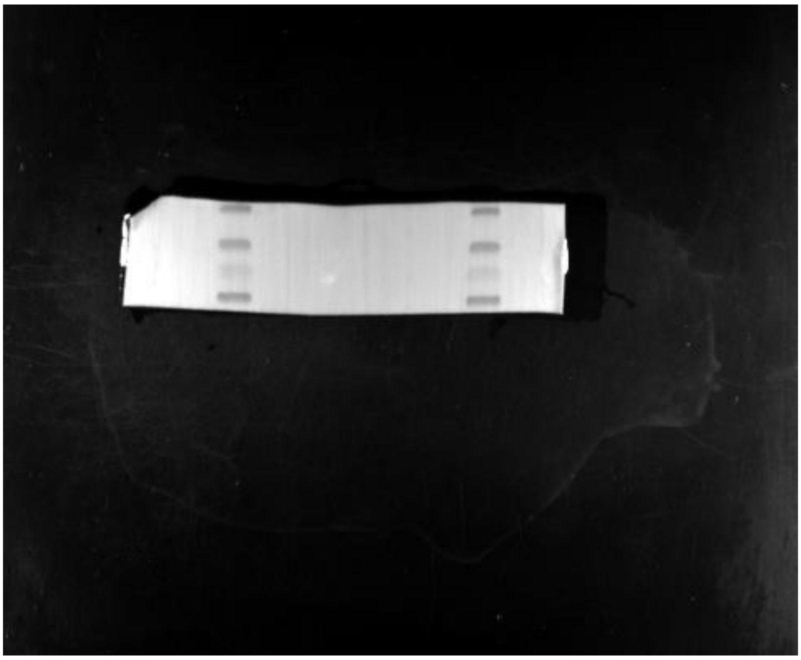

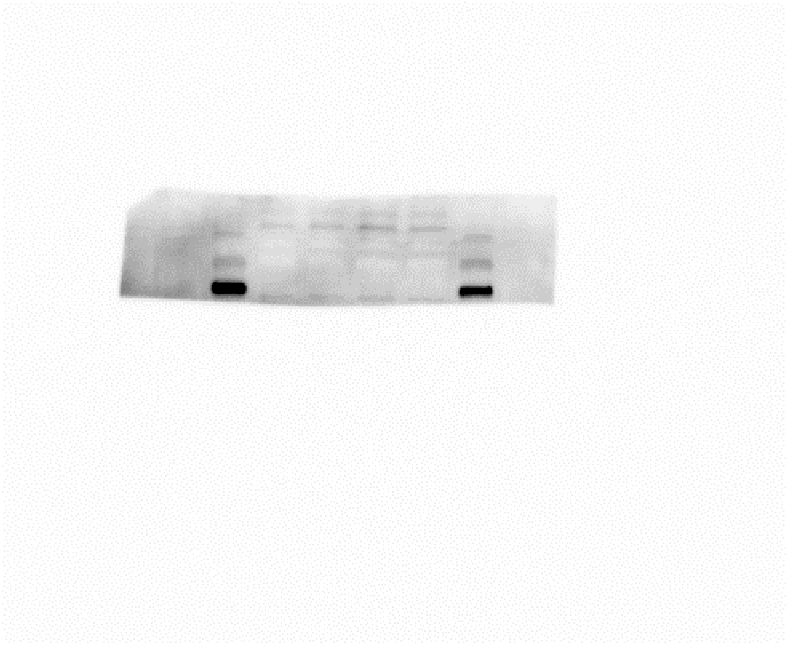

Protein: p-Smad7

Samples of each column from left to right:

1. Arecoline + 100ug/ml ADSC-Exo

2. Arecoline + 50ug/ml ADSC-Exo

3. Arecoline

4. Control

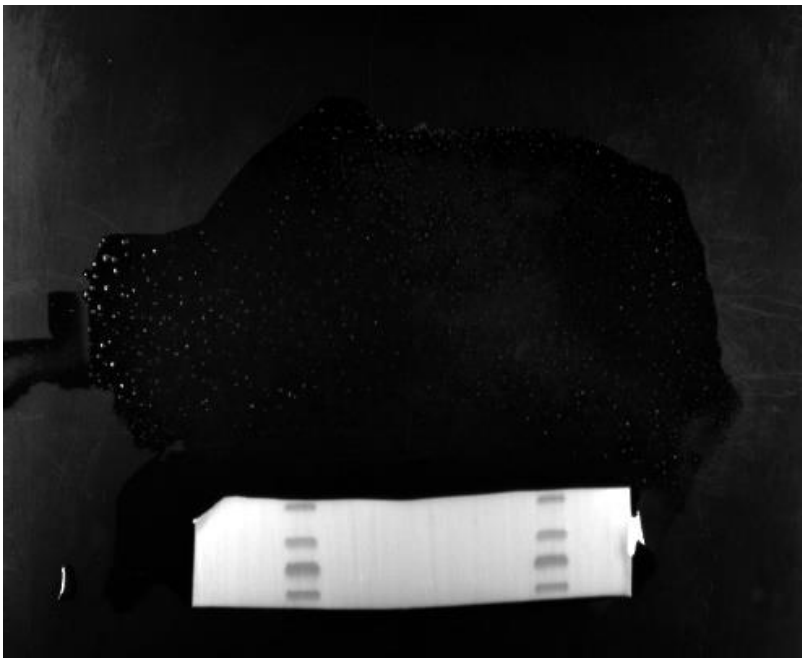

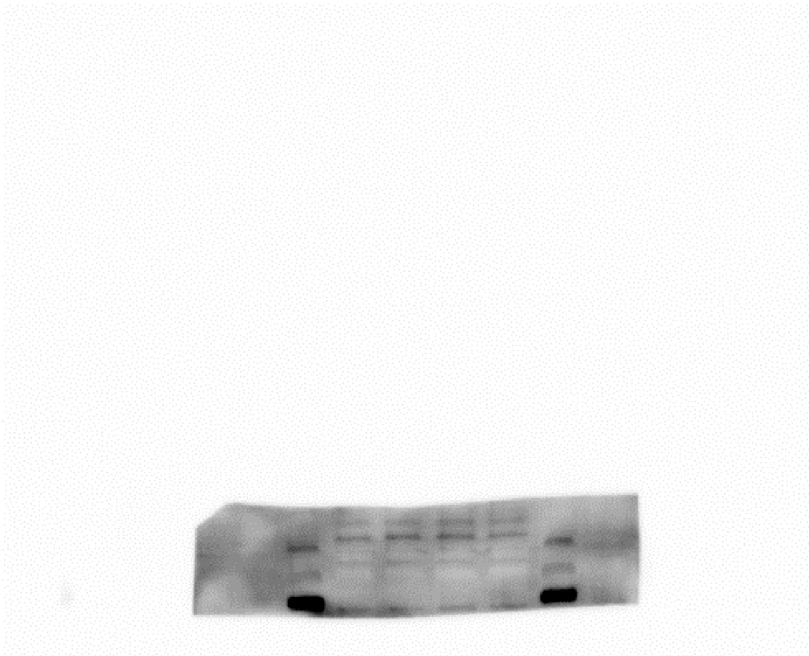

Protein： GAPDH

Samples of each column from left to right：

1. Arecoline + 100ug/ml ADSC-Exo

2. Arecoline + 50ug/ml ADSC-Exo

3. Arecoline

4. Control

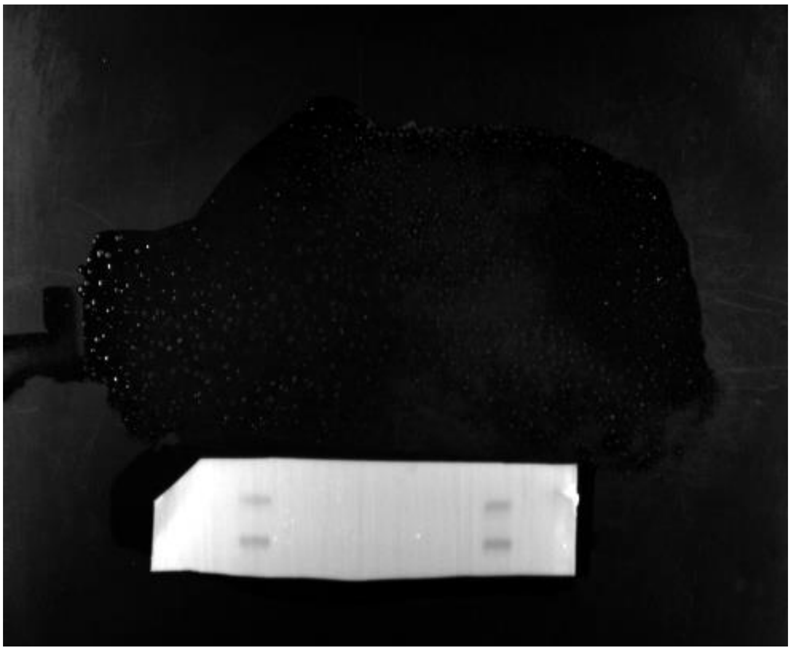

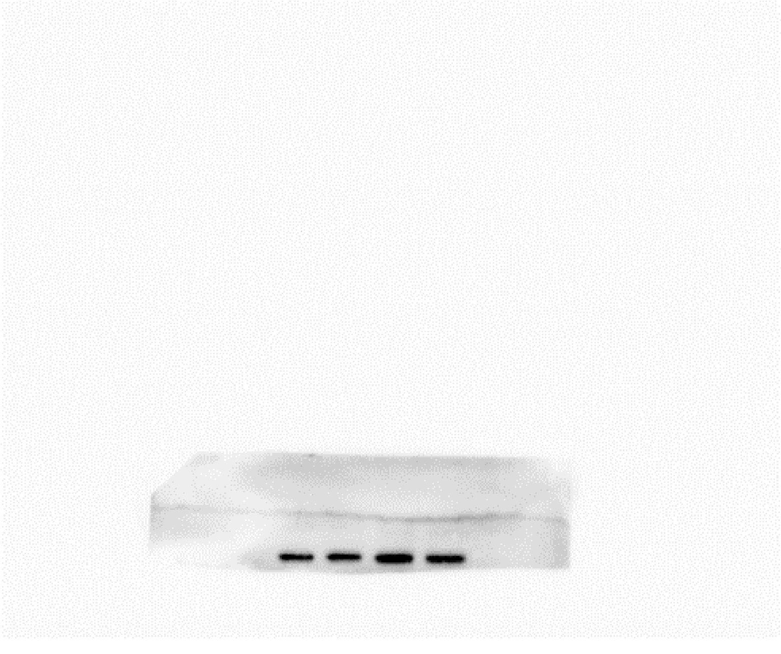

Protein： Smad2

Samples of each column from left to right：

1. Control

2. Arecoline

3. Arecoline + 50ug/ml ADSC-Exo

4. Arecoline + 100ug/ml ADSC-Exo

5. Arecoline + 200ug/ml ADSC-Exo

6. Control

7. Arecoline

8. Arecoline + 50ug/ml ADSC-Exo

9. Arecoline + 100ug/ml ADSC-Exo

10. Arecoline + 200ug/ml ADSC-Exo

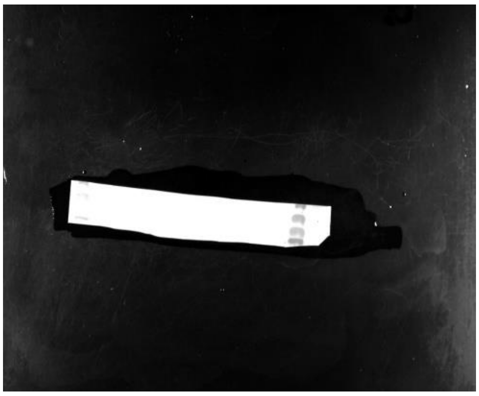

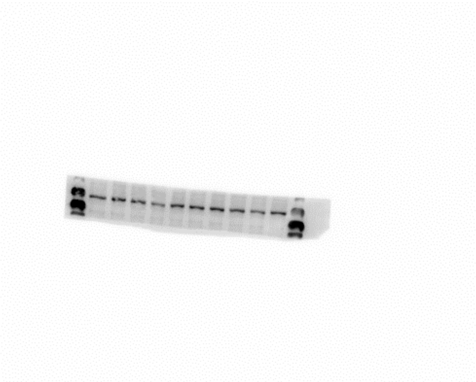

Protein： Smad2/3

Samples of each column from left to right：

1. Control

2. Arecoline

3. Arecoline + 50ug/ml ADSC-Exo

4. Arecoline + 100ug/ml ADSC-Exo

5. Arecoline + 200ug/ml ADSC-Exo

6. Control

7. Arecoline

8. Arecoline + 50ug/ml ADSC-Exo

9. Arecoline + 100ug/ml ADSC-Exo

10. Arecoline + 200ug/ml ADSC-Exo

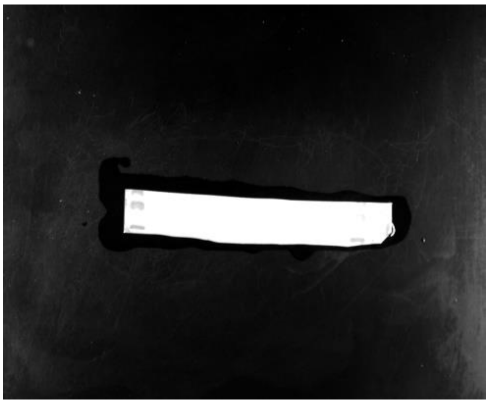

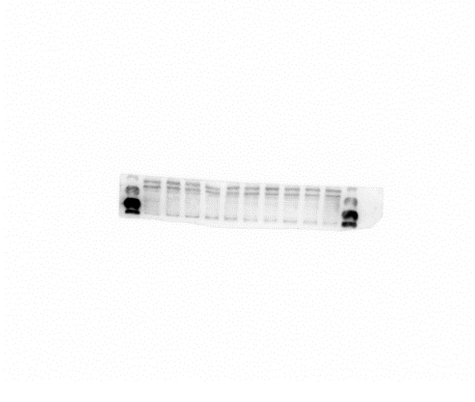

Protein： GAPDH

Samples of each column from left to right：

1. Control

2. Arecoline

3. Arecoline + 50ug/ml ADSC-Exo

4. Arecoline + 100ug/ml ADSC-Exo

5. Arecoline + 200ug/ml ADSC-Exo

6. Control

7. Arecoline

8. Arecoline + 50ug/ml ADSC-Exo

9. Arecoline + 100ug/ml ADSC-Exo

10. Arecoline + 200ug/ml ADSC-Exo

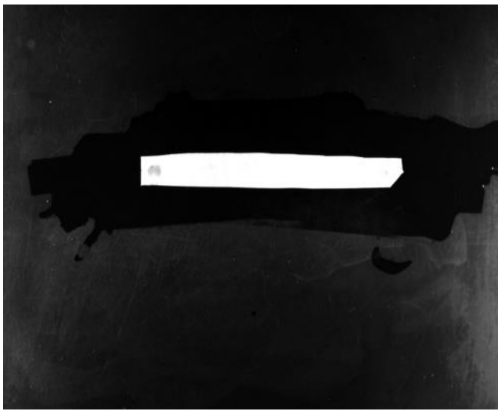

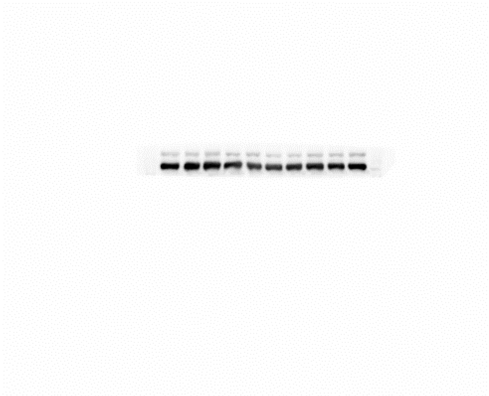

Expression of the Smad2 protein after miR-181a-5p was overexpressed in vitro

Protein:　Smad2

Samples of each column from left to right:

1. mimics NC
2. miR-181a-5p mimics
3. inhibitor NC
4. miR-181a-5p inhibitor

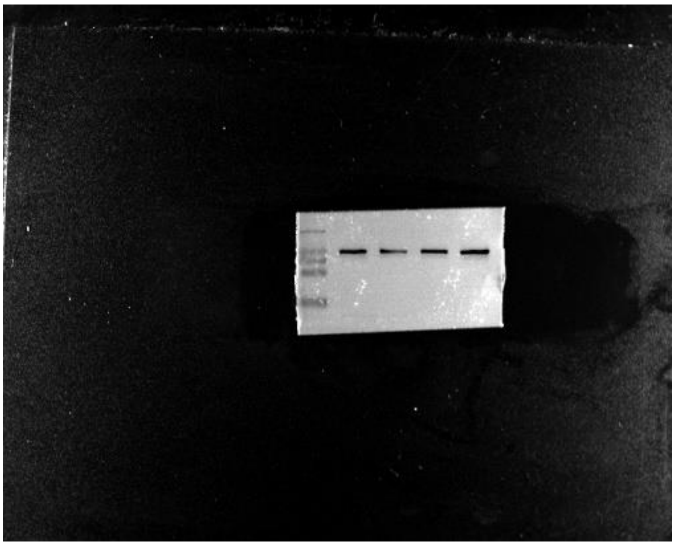

Protein:　GAPDH

Samples of each column from left to right:

1. mimics NC

2. miR-181a-5p mimics

3. inhibitor NC

4. miR-181a-5p inhibitor

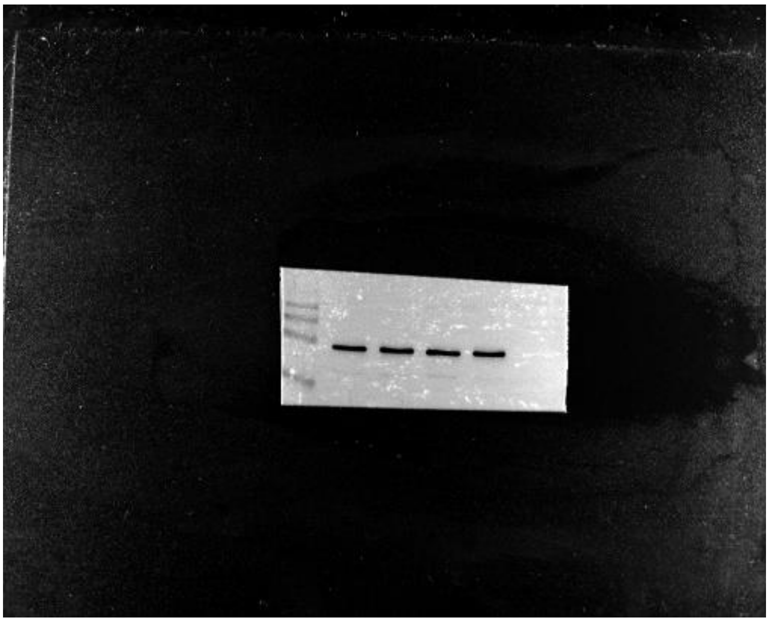

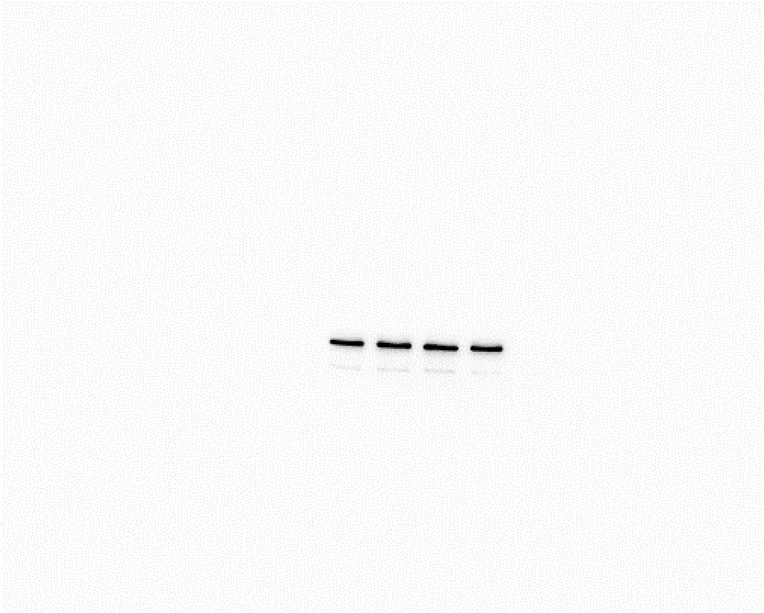

dual-luciferase reporter assay

The qPCR results of wt-psicheck-2-SMAD2 and mut-psicheck-2-

SMAD2

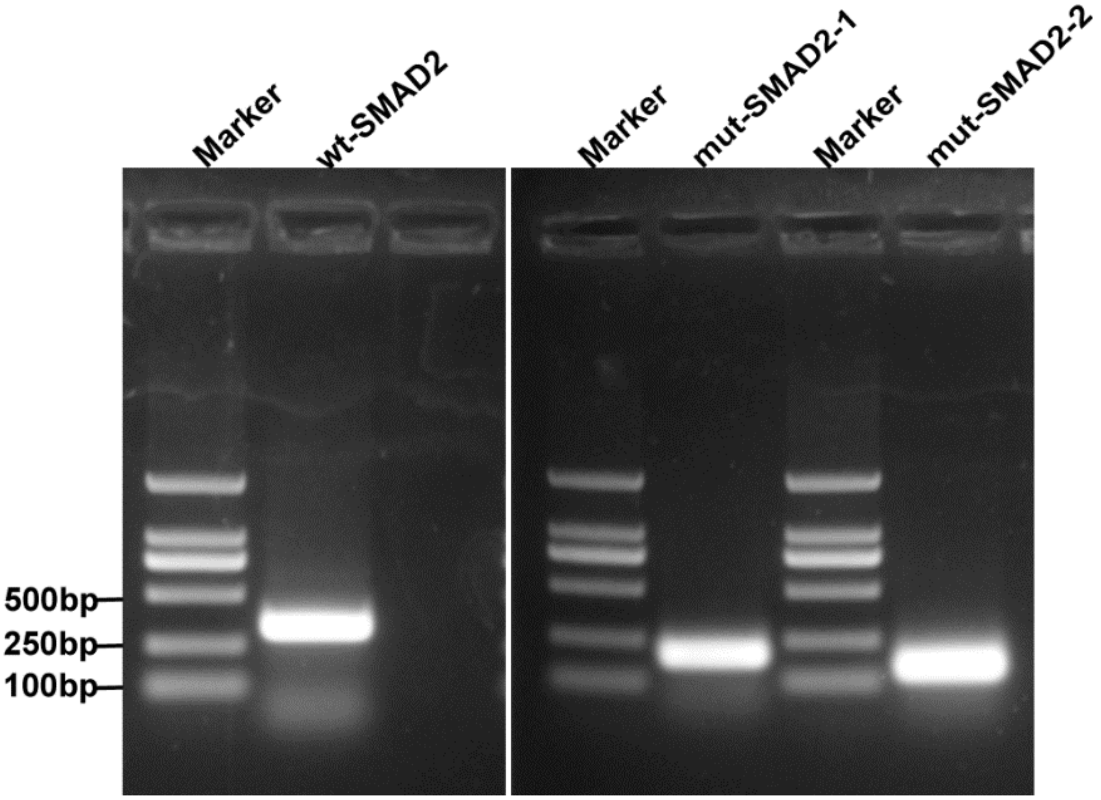

The results were verified by restriction enzyme digestion

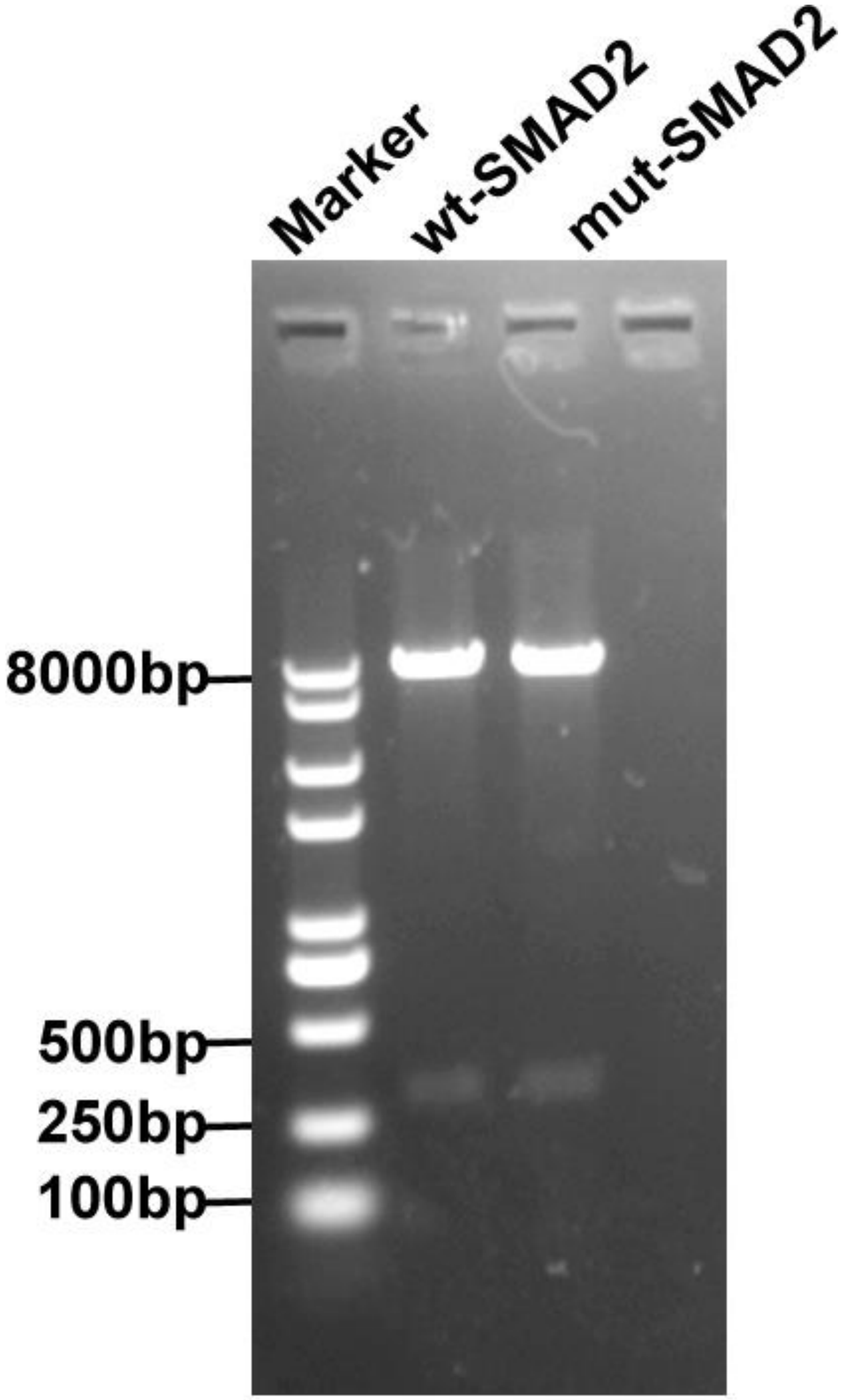

transmission electron microscope (TEM)

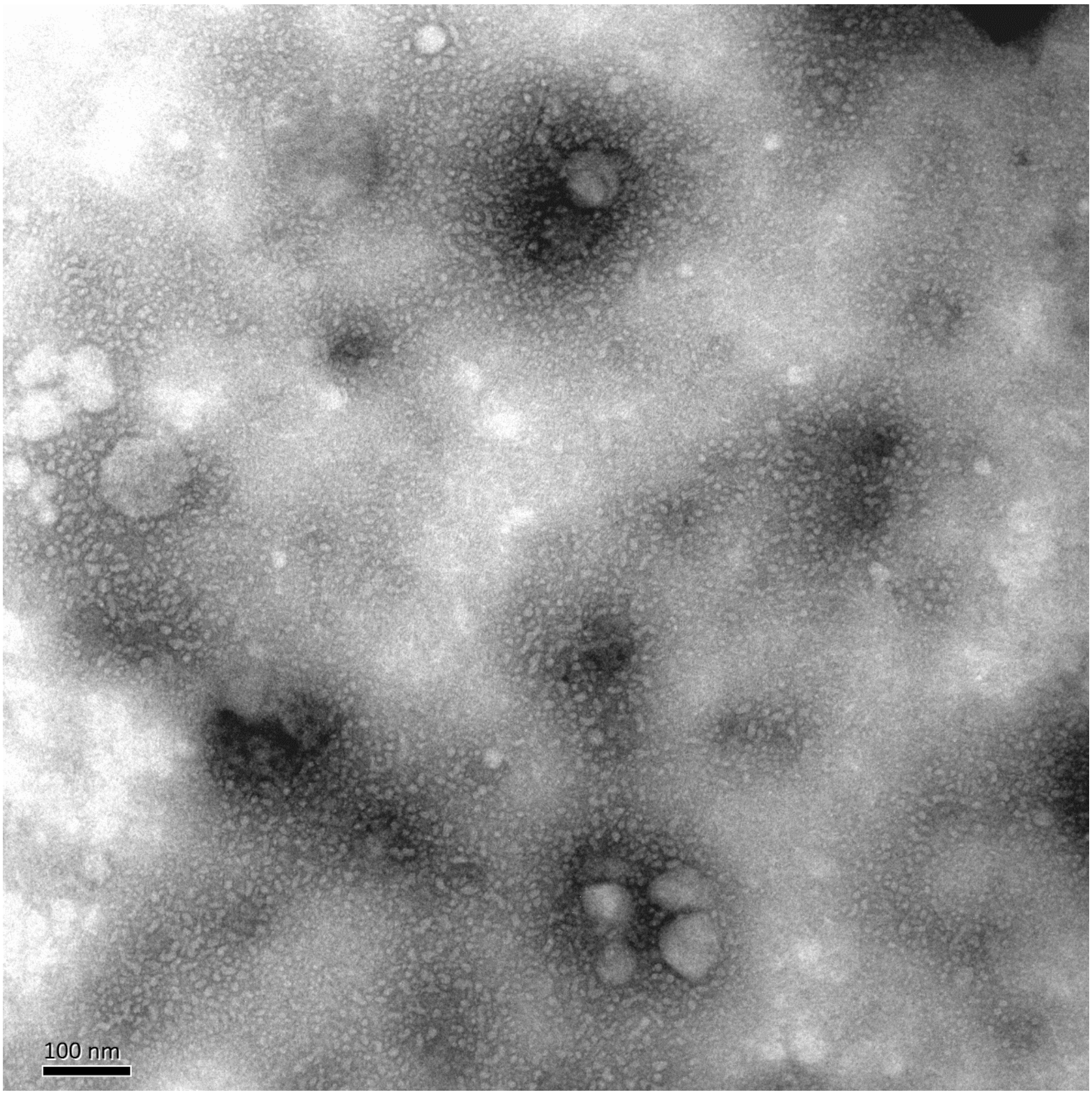

Supplement: Supplementary file 1 — Supplementary file1 (PDF 1513 kb) [file 13770_2023_579_MOESM1_ESM.pdf]
